# Myosins and MyomiR Network in Patients with Obstructive Hypertrophic Cardiomyopathy

**DOI:** 10.3390/biomedicines10092180

**Published:** 2022-09-03

**Authors:** Chiara Foglieni, Maria Lombardi, Davide Lazzeroni, Riccardo Zerboni, Edoardo Lazzarini, Gloria Bertoli, Annalinda Pisano, Francesca Girolami, Annapaola Andolfo, Cinzia Magagnotti, Giovanni Peretto, Carmem L. Sartorio, Iacopo Olivotto, Giovanni La Canna, Ottavio Alfieri, Ornella E. Rimoldi, Lucio Barile, Giulia d’Amati, Paolo G. Camici

**Affiliations:** 1 Cardiovascular Research Center, IRCCS San Raffaele Hospital, 58, 20132 Milan, Italy; 2 Laboratory for Cardiovascular Theranostics, Cardiocentro Ticino Institute, Ente Ospedaliero Cantonale and Faculty of Biomedical Sciences, Università Svizzera Italiana, 6900 Lugano, Switzerland; 3 Institute of Molecular Bioimaging and Physiology, National Research Council (IBFM-CNR), 20054 Milan, Italy; 4 Department of Radiological, Oncological and Pathological Sciences, Sapienza University of Rome, Policlinico Umberto I, 00185 Rome, Italy; 5 Cardiology Unit, Meyer Children’s Hospital, 50139 Florence, Italy; 6 ProMeFa, Proteomics and Metabolomics Facility, IRCCS San Raffaele Scientific Institute, 20132 Milan, Italy; 7 IRCCS San Raffaele Scientific Institute, Vita-Salute University, 20132 Milan, Italy; 8 Cardiomyopathy Unit, Careggi University Hospital, 50134 Florence, Italy; 9 IRCCS Humanitas Clinical and Research Institute, 20089 Rozzano, Italy; 10 Cardiac Surgery Unit, IRCCS San Raffaele Scientific Institute, 20132 Milan, Italy

**Keywords:** hypertrophic cardiomyopathy (HCM), cardiomyocytes, myosin heavy chain (isoform: MyHC, gene: *MYH*), microRNA (*miRs*), MyomiR (myosin-encoded miRs), *miR-499*, SRY-Box Transcription Factor 6 (*SOX6*), Polypyrimidine Tract Binding Protein 3 (*PTBP3*)

## Abstract

Hypertrophic cardiomyopathy (HCM) is the most common genetic cardiomyopathy. The molecular mechanisms determining HCM phenotypes are incompletely understood. Myocardial biopsies were obtained from a group of patients with obstructive HCM (n = 23) selected for surgical myectomy and from 9 unused donor hearts (controls). A subset of tissue-abundant myectomy samples from HCM (n = 10) and controls (n = 6) was submitted to laser-capture microdissection to isolate cardiomyocytes. We investigated the relationship among clinical phenotype, cardiac myosin proteins (MyHC6, MyHC7, and MyHC7b) measured by optimized label-free mass spectrometry, the relative genes (*MYH7*, *MYH7B* and *MYLC2*), and the MyomiR network (myosin-encoded microRNA (*miRs*) and long-noncoding RNAs (*Mhrt*)) measured using RNA sequencing and RT-qPCR. MyHC6 was lower in HCM vs. controls, whilst MyHC7, MyHC7b, and MyLC2 were comparable. *MYH7, MYH7B*, and *MYLC2* were higher in HCM whilst *MYH6*, *miR-208a, miR-208b, miR-499* were comparable in HCM and controls. These results are compatible with defective transcription by active genes in HCM. *Mhrt* and two *miR-499*-target genes, *SOX6* and *PTBP3*, were upregulated in HCM. The presence of HCM-associated mutations correlated with *PTBP3* in myectomies and with *SOX6* in cardiomyocytes. Additionally, iPSC-derived cardiomyocytes, transiently transfected with either *miR-208a* or *miR-499*, demonstrated a time-dependent relationship between MyomiRs and myosin genes. The transfection end-stage pattern was at least in part similar to findings in HCM myectomies. These data support uncoupling between myosin protein/genes and a modulatory role for the myosin/MyomiR network in the HCM myocardium, possibly contributing to phenotypic diversity and providing putative therapeutic targets.

## 1. Introduction

Hypertrophic cardiomyopathy (HCM), the most common genetic cardiomyopathy, is characterized by heterogeneity in both phenotypic expression and clinical course [1]. A variety of mutations predominantly affecting genes encoding for sarcomeric proteins have been demonstrated in HCM [2], and both genetic complexity and epigenetic and/or environmental modifiers may contribute to the phenotype diversity.

Although abnormal expression and altered ratio of the contractility-related myocardial genes and encoded proteins have been previously reported in patients with left ventricular hypertrophy (LVH), dilated cardiomyopathy, and heart failure (HF) [3,4,5,6,7], similar changes have never been described in HCM.

The efficiency of myocardial contraction depends on the activity of cardiac myosin heavy chain protein isoforms (MyHC6, MyHC7, and MyHC7b) [8], whose function is regulated by myosin regulatory light chain 2 ventricular isoform (MyLC2) [4]. The myosin heavy chain genes (*MYH6*, *MYH7*, and *MYH7B*) also encode for intronic microRNAs (*miR-208a*, *miR-208b*, and *miR-499*, belonging to a muscle-specific miRNA family known as MyomiRs). In addition, *Mhrt*, a long non-coding RNA family involved in the development of cardiac hypertrophy, originates from *MYH7* loci [9,10] and a non-coding exon-skipped RNA from *MYH7B* [11]. The interplay between myosins and MyomiRs aimed at controlling myosin content and function, firstly suggested for skeletal muscle, [10,12] has been proposed as a possible common regulatory pathway for striated muscle [10,12]. Changes in the myosin and MyomiR pattern in rodents and on in vitro myocytes have been associated with the suppression of slow fibers, cardiac hypertrophy and dysfunction, and regulation of the myogenic differentiation [13,14,15] via modulation of SRY-Box Transcription Factor 6 (*SOX6*) [16] and Polypyrimidine Tract Binding Protein 3 (PTBP3, also known as ROD1) [17]. The increase in *MYH7* and *MYH7B* expression and/or transcriptional activity is one of the cascade effects associated with *SOX6* inhibition that has been described either in vitro or in animal models. [18,19]. It has been postulated that increased *MYH6* leads to upregulation of *miR-208a*, which in turn inhibits the expression of *SOX6* but promotes the activation/upregulation of *MYH7B*, with subsequent upregulation of *miR-499,* which is another modulator of *SOX6* [20]. Data on if and how this applies to human cardiomyopathies are scant [21], and the extrapolation from animal models to human beings is complicated by distinct expression and twitch characteristics of the myosin isoforms among species [22].

Here we provide a snapshot of the relative expression of MyHCs, MyomiRs, *Mhrt*, and target genes in patients with obstructive HCM. We propose a mechanistic hypothesis explaining the abnormalities of myosins and MyomiRs through the study of how *miR-208a* and *miR-499* may affect the target gene levels over time in human iPSC-derived cardiomyocytes.

## 2. Materials and Methods

### 2.1. Patients

Consecutive patients with obstructive HCM, selected for surgical myectomy at San Raffaele Hospital (Milan, Italy) and Careggi Hospital (Florence, Italy), were prospectively screened and enrolled between December 2014 and December 2018. The study protocol conformed to the ethical guidelines of the 1975 Declaration of Helsinki and was approved by the institutional Ethics committee of both hospitals. All patients signed an informed consent before the study.

Baseline demographic, clinical, and instrumental data (routine ECG and echocardiography) were collected prior to surgery.

#### 2.1.1. Inclusion Criteria

Twenty-seven consecutive patients with obstructive HCM were screened (Figure S1). A total of 4 patients were excluded for technical reasons, and 23 were enrolled. All patients had an unequivocal diagnosis of HCM according to existing guidelines [23], were >18 years of age, in sinus rhythm, with a maximum left ventricular (LV) wall thickness of ≥1.5 cm, the presence of severe resting or inducible LV outflow tract obstruction (peak gradient ≥ 50 mmHg), and a clinical indication for surgical myectomy, i.e., drug-refractory symptoms associated with obstructive HCM.

#### 2.1.2. Exclusion Criteria

Patients were excluded if they had documented coronary artery disease (confirmed by coronary angiography prior to surgery), valvular aortic stenosis, uncontrolled arterial hypertension, Fabry disease or other HCM mimics.

### 2.2. Control Hearts

Left ventricle myocardial biopsies from unused donor hearts collected at Policlinico Umberto I Hospital (Rome, Italy) served as controls (CTRLs). Since Italian privacy law protects information about donors, data about these subjects were not available. Control biopsies were extensively sampled and examined under light microscopy to exclude the presence of cardiomyocyte hypertrophy, necrosis or degenerative changes, significant fibrosis, or inflammatory infiltrates. Two additional control samples, an atrial fragment from a heart transplant recipient with dilated cardiomyopathy and a biopsy from an unaltered deltoid muscle, were similarly collected and used as controls in mass spectrometry (MS) analysis (see 2.7).

### 2.3. Sample Processing

Biopsies collected during surgery were cut into 2 mm thick slices perpendicularly to the endocardium. Slices were snap-frozen in liquid nitrogen for protein and mRNA extraction. Serial cryosections (10–20 μm thick) were obtained by a Leica CM1850 cryostat (Leica Microsystems GmbH, Wetzlar, Germany), collected on slides or in vials for subsequent use. One additional slice was fixed in a 10% neutral buffered formalin solution (Sigma-Aldrich, Merck, Darmstadt, DE, Germany) and processed for conventional paraffin inclusion, sectioned at 5 μm of thickness and used for histologic evaluation.

Blood samples (10 mL) from patients with HCM and from a group of healthy volunteers (n = 11), matched for age and gender, were withdrawn in EDTA tubes and processed to obtain plasma.

### 2.4. Genetic Testing

Total DNA was extracted from at least 20 cryosections using the DNeasy Blood & Tissue kit (Qiagen, Hilden, Germany). Genetic analysis was performed by next-generation sequencing on a MiSeq platform using the TruSight Cardio Sequencing kit (Illumina, San Diego, CA, USA). A panel of 26 HCM-associated genes was analyzed (Table S1). Read alignment, variant calling and annotation were performed using the MiSeq Reporter and the Variant Interpreter software (Illumina, San Diego, CA, USA). All variants reported in this work were validated by Sanger sequencing and classified as pathogenic, likely pathogenic, or of uncertain significance following the latest variant interpretation guidelines [24] using Cardio-Classifier or InterVar (for variants in genes that Cardio-Classifier does not analyze) [24,25,26].

### 2.5. Histology and Morphometry

Sections (5 μm thick, each at 100 μm distance) were taken from paraffin samples, dewaxed, hydrated, stained with hematoxylin/eosin or Azan-Mallory trichrome, dehydrated and mounted. Blinded analysis was performed.

### 2.6. ELISA

The amount of cardiac MyHC7b was quantified in myocardial protein extracts by a specific ELISA (MyBioSource Inc., San Diego, CA, USA) following the manufacturer’s instructions. Luminescence measurement was performed on an Infinite F200 microplate reader (TECAN Group Ltd., Männedorf, Switzerland). Samples were run in triplicate.

### 2.7. MS Analysis of Myosin Proteins

Protein extracts enriched for myosins were obtained from myocardial cryosections by incubation with freshly prepared extraction buffer (300 mM NaCl, 0.1 M NaH_2_PO_4_ monohydrate, 50 mM Na_2_HPO_4_ anhydrous, 10 mM sodium pyrophosphate, 1 mM MgCl_2_, 10 mM EDTA, and fresh 1.4 mM 2-mercaptoethanol, pH 6.5) in a 1:4 m/v ratio for 1 h on ice over an orbital shaker. The extracts were centrifuged at 12,000× *g* for 10 min at 4 °C, and the supernatants were diluted 1:1 *v*/*v* in conservation buffer (40 mM sodium pyrophosphate and 50% glycerol in distilled water, pH 8.5). Proteins were dosed by standard Bradford assay, aliquoted, and stored at −80 °C until use [27,28]. In the optimization phase, enriched and standard extractions were carried out in parallel on two sets of tissue samples from 1 HCM patient, 1 CTRL, 1 normal human atrium from a donor heart, and 1 skeletal muscle biopsy with no evidence of morphological alterations (positive control, for enrichment only). Equal amounts of proteins (15 µg/lane) were loaded on precast 4–12% SDS PAGE gel (Invitrogen, Carlsbad, CA, USA) and run in MOPS buffer in non-reducing conditions. Coomassie-stained bands were compared, assessing the intensity of the band at the myosin molecular weight range (Figure S3). The bands, including proteins at the molecular weight of myosins (see arrows in Figure S3), were reduced, alkylated and digested as previously described [29]. Aliquots of the sample were de-salted, re-suspended in 12 µL 10% formic acid and analyzed by nLC-ESI-MS/MS using a Q-Exactive (Thermo Fisher Scientific Inc., Waltham, MA, USA) equipped with a nano-electrospray ion source (Proxeon Biosystems, Odense, Denmark). Peptides were separated in a capillary chromatographic system (3 µL injected, EASY-nLC 1000 Integrated Ultra High-pressure Nano-HPLC System, Proxeon Biosystem, Odense, DK) using a 15 cm reverse phase silica capillary column packed with 1.9 μm ReproSil-Pur 120 C18-AQ. A 60 min-gradient was applied to achieve separation (0.30 μL/min flow rate) using water with 0.1% formic acid and acetonitrile with 0.1% formic acid. The ten most intense doubly and triply charged ions were selected and fragmented in the ion trap. All MS/MS data were analyzed using the Mascot software (version 2.6, Matrix Science) search engine to search the UniProt_Human Complete Proteome_cp_hum_2019_0213 (95,943 sequences; 38,082,498 residues).

Searches were performed with 2 missed cleavages allowed, carbamidomethylation on cysteine as fixed modification, protein N-terminus-acetylation and methionine oxidation as variable modifications, and +2 and +3 peptide charge. Mass tolerance was set to 5 ppm and 0.02 Da for precursor and fragment ions, respectively. The resulting Mascot score is a statistical score for how well the experimental data match the database sequence. The Mascot score for a protein is the summed score for the individual peptides, including peptide masses and peptide fragment ion masses, for all peptides matching a given protein. For positive protein identification, the Mascot score must be above the 95% confidence level. The Mascot score is a logarithmic score and is described in detail on the Matrix Science website (http://www.matrixscience.com/).

Upon enrichment, protein extracts (20 µg/sample) underwent in solution reduction in 10 mM dithioerythritol diluted in 0.1 M NH_4_HCO_3_ (1 h, 56 °C, shaking), then alkylation with 27 mM iodoacetamide diluted in 0.1 M NH_4_HCO_3_ (30 min, room temperature in the dark) and digestion with trypsin 1:50 *v/v* (37 °C, overnight, shaking). The tryptic mixture was de-salted and re-suspended in 20 µL 10% formic acid. Peptides were separated as described above. A 100 min-gradient was applied to achieve separation (0.30 μL/min flow rate) using water with 0.1% formic acid (sol A) and acetonitrile with 0.1% formic acid (sol B). In detail, after 5 min elution at 2% acetonitrile in sol B, its percentage was gradually increased up to 40% in 83 min, followed by 5 min of washing in 90% acetonitrile.

Parallel reactions monitoring (PRM) label-free targeted proteomic approach, optimally suited for relative quantification of proteins across samples [30], was performed by a Q-Exactive mass spectrometer. The acquisition method combined two scan events corresponding to a full scan MS from 300 to 2000 m/z (resolution setting of 35,000 at m/z 200) and a PRM event (resolution setting of 17,500 at m/z 200; isolation window set to 2 m/z; maximum fill time of 120 ms and normalized collision energy set to 27), which targeted the precursor ions of the peptides at their relevant charge states in a 1 min window. Using trypsin-digested proteins, a local spectral library was created to generate reference MS/MS spectra (b- and y fragment ions) and to determine the elution time for each myosin peptide. Peptides (8–16 amino acids long) were selected for PRM measurements based on their unique occurrence in a single myosin isoform and their presence in the PeptideAtlas [31] in most cases. Artificial modification sites (like methionine or cysteine residues) as well as protein sequence regions containing mutations for this population study were avoided. Data processing was performed with Skyline 4.2.0.19072 software (64-bit) [32]. Post-acquisition quantification was performed by extracting the chromatographic traces of specific fragment ions (from 3–6 transitions). Peak areas calculated as precursors (MS1 total area) and from the sum of transitions (MS2 total area) were automatically estimated by the Skyline software, providing relative quantification of myosin isoforms. Four technical replicates/samples were run.

The atrium and skeletal muscle samples [33] (positive controls for the MyHC6 and MyHC7 and negative control for MyHC7b and ventricular MyLC2 proteins) served as comparators and confirmed the reliability of the data acquired by the PRM method.

### 2.8. Laser-Capture Microdissection and RNA Extraction

A subset of tissue-abundant myectomy samples from HCM patients (n = 10) and CTRLs (n = 6) was serially cryosectioned (8-μm-thick sections) and submitted to laser-capture microdissection (LCM). Briefly, the sections were mounted on polyethylene naphthalate membrane frame slides and stained by hematoxylin/eosin. Small groups of cardiomyocytes from both morphologically organized and disorganized regions were microdissected in a balanced amount using an MMI NIKON UVCUT System (20X objective, NIKON, Tokyo, Japan) equipped with an ultraviolet laser. Coronary arteriole-containing interstitial areas were also sampled. Samples were collected by gravity into microcentrifuge tubes filled with RNAlater Stabilization Solution (ThermoFisher Scientific Inc., MA, Waltham, USA) for successive RNA extraction [34]. Total RNA was isolated by an miRNeasy Micro Kit (QIAGEN, Manchester, UK). Synthesis of cDNA and RT-qPCR were performed (see 2.11).

### 2.9. Generation of Human Induced Pluripotent Stem Cells, Differentiation to Cardiomyocytes and Transfection

The induced pluripotent stem cells (iPSC) were obtained by the reprogramming of adult stromal cardiac-specific mesenchymal cells (cMSC9 cells from a biobank, established within previous studies at Cardiocentro Ticino Institute_Ente Ospedaliero Cantonale) [35]. Studies on iPSC were approved by the Comitato Etico Cantonale, Bellinzona, Switzerland (Ref. CE 2923) and performed according to the Declaration of Helsinki.

The cMSC were transduced with the integration-free Sendai virus cocktail hKOS:hc-Myc:hKlf4 at an MOI of 5:5:3 (CytoTune-iPS 2.0 Sendai Reprogramming Kit, Thermo Fisher Scientific Inc., Waltham, MA, USA), as per the manufacturer’s instructions. Seven days after transduction, the medium was changed to StemFlex (Thermo Fisher Scientific Inc., Waltham, MA, USA). Individual colonies with embryonic stem cell-like morphology were transferred into 12-well plates coated with Matrigel (hESC Qualified Matrix, Corning, NY, USA) and iPSC expanded. Differentiation into cardiomyocytes was performed through the StemMACS CardioDiff Kit XF (Miltenyi Biotec, Bergisch Gladbach, Germany) following the manufacturer’s protocol. Metabolic selection of cardiomyocytes started on day 10 and ended on day 17 of differentiation and was performed in RPMI1640 without glucose (Thermo Fisher Scientific Inc., Waltham, MA, USA) additioned with 0.5 mg/mL human recombinant albumin, 0.2 mg/mL L-ascorbic acid 2-phosphate, and 4 mM lactate (Sigma-Aldrich, Merck, Darmstadt, DE, Germany). Afterwards, cardiomyocytes were cultured in a maintenance medium until at least day 30 for further maturation; then, *mir*Vana miRNA mimics (Ambion, Thermo Fisher Scientific Inc., Waltham, MA, USA) either for *miR-499* or *miR-208a* were delivered by Lipofectamine RNAiMAX Transfection Reagent (Thermo Fisher Scientific Inc., Waltham, MA, USA). After 24 h, new medium was added, and after a further 24 h, the medium was replaced with serum-free medium (DMEM High Glucose, GIBCO), and cardiomyocytes were cultured [36].

### 2.10. Extracellular Vesicles Isolation

Extracellular vesicles (EV) were isolated from a subset of HCM patients (n = 12) and healthy volunteers (n = 8) plasma samples and from iPSC-derived cardiomyocyte supernatants [37]. The purity of EV from both plasma and supernatant samples was tested by Western blotting in reducing conditions [36]. Total protein extracts (20 µg) were loaded on an 8–20% acrylamide gel, then transferred to a PVDF membrane and incubated in Intercept blocking buffer for 1 h at room temperature (LI-COR Biosciences, Lincoln, NE, USA) followed by primary antibodies against ALG-2 interacting protein X (Alix, part of Endosomal Sorting Complexes Required for Transport [38], ab186429, Abcam, Cambridge, UK), tumor susceptibility gene 101 (TSG101, an ESCRT component [39], ab30871 Abcam, Cambridge, UK) and tetraspanin CD81 [40] (MA5-17937, Thermo Fisher Inc., Waltham, MA, USA). After washing, the membranes were incubated for 1 h with secondary antibody (anti-mouse IgG IRDye-conjugated, LiCor). Images were acquired by Odyssey DLx and analyzed by Image Studio software (LI-COR Biosciences, Lincoln, NE, USA).

### 2.11. RNA Extraction: Gene and miRNA Expression Analysis

The mRNA, including miRNA-enriched fraction, was isolated from snap-frozen tissue sections (at least 20 sections, 10 μm thick), LCM samples, plasma [41] and EV using a miRNeasy mini kit and a RNeasy mini kit (Qiagen, Hilden, DE). Synthesis of cDNA was carried out either by a High Capacity RNA-to-cDNA Kit for mRNA or by aTaqMan MicroRNA reverse transcription kit for miR (Invitrogen, Carlsbad, CA, USA), respectively. The RT-qPCR was performed using TaqMan Universal Master Mix II. Taqman primer/Fam-labeled probes (Hs01101429_g1, Hs01110593_g1, Hs00936877_g1, Hs00264525_m1, Hs01006016_m1 and Hs0494234_g1, Applied Biosystems, Foster City, CA, USA) determined the relative levels of *MYH6*, *MYH7*, *MYH7B*, *SOX6*, *PTBP3* and *Mhrt*, respectively. Taqman Fam-labeled probes (*hsa-miR-499-5p*, *hsa-miR-208a-5p* and *hsa-miR-208b-5p*, Applied Biosystems, Foster City, CA, USA) determined the relative levels of *miR-499*, *miR-208a*, and *miR-208b*. All the procedures were carried out according to the manufacturer’s instructions. Samples were run in triplicate. Target gene levels were normalized to that of *β-actin* (Hs01060665_g1) and miR levels to that of *hsa-miR-103a-3p* (chosen for its stability). Relative expression was determined using the ΔCt method [42]. Total RNA, including miRNA-enriched fraction, was obtained from plasma using miRNeasy Serum/Plasma kit (Qiagen, Hilden, Germany). Synthesis of cDNA and RT-qPCR for *miR-499, miR-208a*, and *miR-208b* were performed as previously described [41]. An independent cardiac sample inserted in all the experiments allowed for the evaluation of the intra-plates’ variability (<5%), and it was used for sample normalization.

RNA from EV (200 ng) was retro-transcribed in cDNA with a TaqMan MicroRNA Reverse Transcription Kit (Invitrogen, Carlsbad, CA, USA) following the manufacturer’s protocol, and cDNA (20 ng) was used as a template for the digital droplet PCR (ddPCR) assay loaded on DG8 cartridge (Bio-Rad, Cressier, Switzerland) with the opportune specific probe (see above) diluted in ddPCR Supermix for Probes (Bio-Rad, Hercules, CA, USA). Obtained droplets were amplified with PCR reaction following the manufacturer’s protocol. Fluorescence was then acquired and analyzed by a QX100 droplet reader equipped with QX manager software (Bio-Rad, Hercules, CA, USA).

### 2.12. RNA Sequencing

The mRNA (RIN > 6, determined by Agilent RNA 6000 Pico Assay using Agilent 2100 Bioanalyzer, Santa Clara, CA, USA) from samples obtained by LCM was submitted to transcriptome profiling with Illumina Nextseq 500 with a HighOutput flow cell, 1x75nt, single read. Sequencing libraries were prepared using a Clontech Smarter kit. FastQC software was used to examine the quality of fastq files (http://www.bioinformatics.babraham.ac.uk/projects/fastqc). Trimmed sequences were aligned to the mm10 genome by STAR [43]. Read counts were calculated as RPKM (reads per kilobase per million) and presented as log2 (RPKM).

### 2.13. In Silico Analysis

The prediction of miRNA targets and protein networks was performed by miRDB [44] and STRING v11 [45] online databases, respectively.

### 2.14. Statistical Analysis

Either the Shapiro–Wilk or the D’Agostino and Pearson omnibus normality test was applied to assess the normality of data distribution, as appropriate. Data from HCM and CTRL samples were compared by Mann–Whitney test, *t*-test or Kruskal–Wallis test with Dunn’s Multiple Comparison posthoc test. Comparison among clinical and experimental variables was performed by Spearman correlation or by logistic probability analysis. ANOVA with/without Friedman’s test for multiple comparisons was applied to evaluate the cell transfection effects on expressed genes. Probability values of <0.05 were considered statistically significant. Prism 8.2 software was used.

## 3. Results

### 3.1. Baseline Characteristics of Study Patients

Surgical samples from 23 patients with obstructive HCM (flowchart of the study in Figure S1) were harvested. None of the study patients were in a high-risk category at the time of surgery. Patient demographic and baseline clinical characteristics are reported in Table 1.

The average maximum wall thickness value was 21 mm (range 15–38 mm), and the mean peak LV outflow gradient was 80 mmHg (Table 2). All patients had normal or supernormal LV ejection fraction (mean 67 ± 9%) in the presence of a non-dilated LV. Genetic testing demonstrated the presence of pathogenic/likely pathogenic variants in HCM-associated sarcomeric genes (listed in Table S1) in 40% of the patients (Table S2).

For the CTRLs, myectomy samples from 9 unused donor hearts were obtained.

### 3.2. Histology

The myectomies from HCM patients showed variable extents of myofiber hypertrophy and disarray, interstitial fibrosis, micro-scarring and remodeling of intramural coronary arterioles, consistent with the clinical diagnosis of HCM (Figure S2). None of the HCM samples showed histologic features of storage disease. All sections from CTRL demonstrated features compatible with preserved healthy hearts.

### 3.3. Myosin Isoforms in HCM Myocardium: Protein Analysis

The homology between MyHC6 and MyHC7 protein sequences (93%, due to the tandem chromosomal link of *MYH6* and *MYH7* genes) and between either of them and MyHC7b (≈70%, encoded on a different chromosome) hampers a wide application of antibody-based methods to discriminate between isoforms. Commercial ELISA kits specific for MyHC7 and MyHC7B without expected cross-reactivity with homologue isoforms were identified (Figure S3A) and tested on myectomy extracts, showing no significant difference between HCM and CTRL samples (Figure S3B). No suitable ELISA was found for MyHC6 (interference levels exemplified in Figure S3A).

To validate ELISA data and also evaluate MyHC6 in the myocardial samples, a highly specific PRM targeted proteomic approach was applied using MS. Preliminary experiments demonstrated that introducing a procedure for tissue extracts enrichment [27,28] increased the myosin electrophoretic bands (Figure S4A). The analysis of the MS results by Mascot software v2.6 attributed the highest score to the myosin family for the 191 kDa band and to MyLC2 for the 19 kDa band (Figure S4B). The proteotypic peptides (i.e., peptides showing unique presence in a single myosin chain isoform) selected for PRM measurements of MyHCs and MyLC2 based on their unique presence in a single myosin chain isoform were clearly detectable in myectomy extracts upon enrichment (see representative PRM chromatographic traces for proteotypic MyH7b peptide in Figure S4C and the corresponding sequences in Figure S4D).

**Figure 1:**
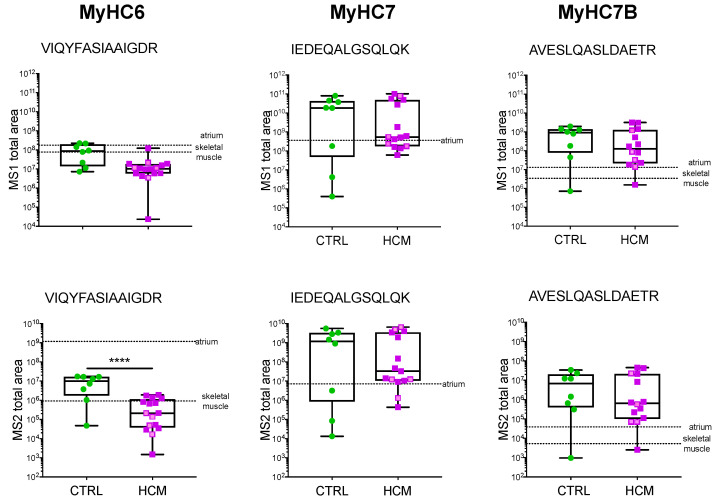
**Myosin protein analysis by MS.** PRM quantification of myosin isoforms in HCM (n = 14) and CTRL (n = 8) is presented as MS1 and MS2 total area of representative selected peptides. The HCM patients without HCM-associated mutations are presented in violet, and those bearing mutations are presented in pink. Two additional morphologically normal human samples, from atrium and skeletal muscle biopsies, serve as comparators (dotted lines). Mean values from technical replicates/samples are presented as dots and boxes (min to max). Unpaired *t*-test was applied; significant differences with *p* < 0.0001 are indicated by ****.

PRM showed that MyHC6 was significantly less abundant in HCM vs. CTRL, both at precursor (MS1 total area, *p* = 0.0023) and fragment levels (MS2 total area, *p* = < 0.0001) (Figure 1). No significant difference between HCM and CTRL in the expression level of MyHC7, MyHC7b, and MyLC2 was detected (Figure 1 and Figure S4E).

No relationship between myosin isoform expression level by PRM and mutations or clinical data was detected.

### 3.4. Myosin Isoforms in HCM and MyomiR Network in the Myocardium: Gene Expression Analysis

The lower levels of MyHC6 in HCM compared to CTRLs, in the absence of compensative changes in MyHC7b or MyHC7 and of differences in MyLC2, could indicate either a transcriptional defect or altered gene expression. To ascertain whether *MYH6*, *MYH7*, *MYH7B*, and *MYLC2* gene expression paralleled the encoded proteins, the mRNA expression levels were determined in myectomies by RT-qPCR.

**Figure 2:**
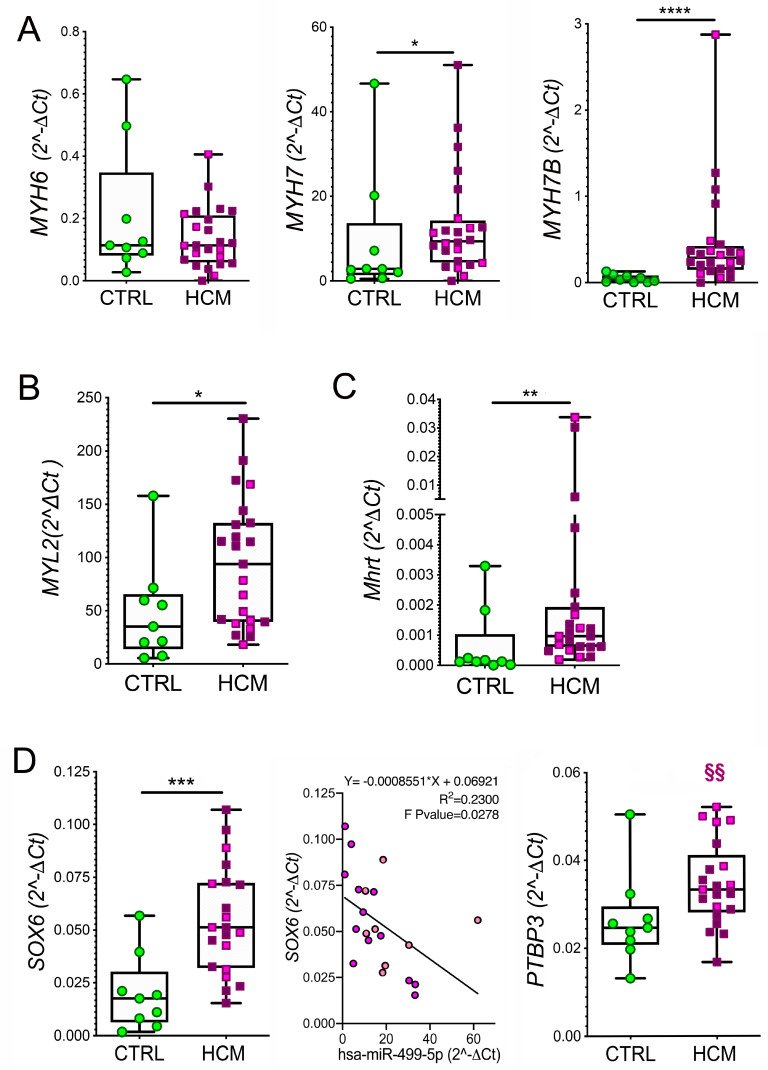
**MyomiR network gene expression in myectomies: myosin genes, Mhrt and *miR-499* targets.** The expression levels of *MYH6, MYH7*, and *MYH7B* mRNA (**A**), of *MYLC2* mRNA (**B**) and of *Mhrt* (**C**) evaluated by RT-qPCR in the myocardium of HCM vs. CTRL are shown. Values are presented as boxes (min to max), and dots indicate single sample values. The expression levels of *miR-499*-target genes coding for transcription factors, *SOX6* ((**D**)**, left and middle panels**) and *PTBP3* ((**D**)**, right panel**) by RT-qPCR are compared in the myocardium of HCM and CTRL. Values are presented as boxes (min to max), and dots indicate single sample values. The inverse relationship between *miR-499* and *SOX6* is plotted ((**D**)**, middle**, **panel**). The HCM patients without HCM-associated mutations are presented in violet; those bearing mutations are in pink. Unpaired *t*-test is applied and significant differences are shown as * *p* < 0.05; ** *p* < 0.01 and *** *p* < 0.0005, **** *p* < 0.0001. Significance within HCM group between individuals with or without mutations is indicated as §§ *p* < 0.01.

In contrast to protein levels, no appreciable difference in *MYH6* expression between HCM and CTRL was found. The expression levels of *MYH7, MYH7B* (*p* = 0.0130 and *p* < 0.0001, respectively, Figure 2A), and *MYLC2* (*p* = 0.0471, Figure 2B), as well as the *MYH7/MYH6* and *MYH7B/MYH6* ratios (*p* = 0.0384 and *p* < 0.0001, respectively, Figure S5A) were significantly higher in HCM compared to CTRL. The cardiac-specific splicing product of *MYH7*, *Mhrt*, was also upregulated in HCM vs. CTRL (*p* = 0.0130, Figure 2C), and its level correlated with that of *MYH7* (Figure S5B*)*. The expression level of these RNAs was unrelated to mutations in HCM (Figure 2A–C).

The expression of *MYH*-encoded MyomiRs, i.e., *miR-208a*, *miR-208b* and *miR-499*, was comparable in HCM and CTRL myectomies (Figure S5C); did not parallel the increase in *MYHs* in HCM samples; and was unrelated to patient mutations. These data possibly indicate defective *MYH* transcription in HCM or MyomiR release into the bloodstream.

### 3.5. Expression of miR-499 Targets in the Myocardium: SOX6 and PTBP3

To verify the putative importance of *SOX6* and *PTBP3* in humans, in silico analysis was performed. Among the 591 putative target genes for *miR-499* found in humans via the MiRDB mining engine, a 100% score was attributed to *SOX6* and an 89% score to *PTBP3* (Supplementary Materials S1). Further analysis by STRING v11 showed that *SOX6* and *PTBP3* encode for transcription factor proteins co-expressed with putative homologs of the myosin isoforms in species different from *Homo sapiens*, but it did not provide data on co-expression links with myosins in humans (Figure S6).

The investigation of the myocardium by RT-qPCR showed significantly higher expression of *SOX* in HCM than in CTRL (Figure 2D left). A moderate negative linear relationship was found between *SOX6* and *miR-499* (Figure 2D middle), in agreement with the inhibitory function of *miR-499*. Moreover, *PTBP3* was not different in HCM vs. CTRL, but significantly high expression of *PTBP3* was related to the presence of mutations in HCM (*p* = 0.0060; Figure 2D, right).

### 3.6. Myosins and MyomiR Network in Cardiomyocytes from Myectomies

To investigate whether the above results seen in the HCM myocardium were affected by abnormal expression of genes by cells other than cardiomyocytes (e.g., by myofibroblasts or dedifferentiated vascular smooth muscle cells), a subset of myectomy tissues was submitted for LCM microdissection. RNA sequencing demonstrated a greater relative abundance of expressed *MYH6, MYH7,* and *MYH7B* (*p* = 0.0001, *p* < 0.0001, and *p* = 0.0007, respectively; Figure S7A, left) in the HCM cardiomyocyte areas compared to coronary arteriole-containing interstitial areas. RT-qPCR confirmed these differences (Figure S7B) and demonstrated that expression levels of *MYHs* were comparable in the myocardial tissue and cardiomyocyte samples (Figure S7B). RNA sequencing also showed a prevalence of *SOX6* (*p* = 0.0004) and *PTBP3* (*p* < 0.0001) transcription factor genes outside cardiomyocytes (Figure S7A, right).

**Figure 3:**
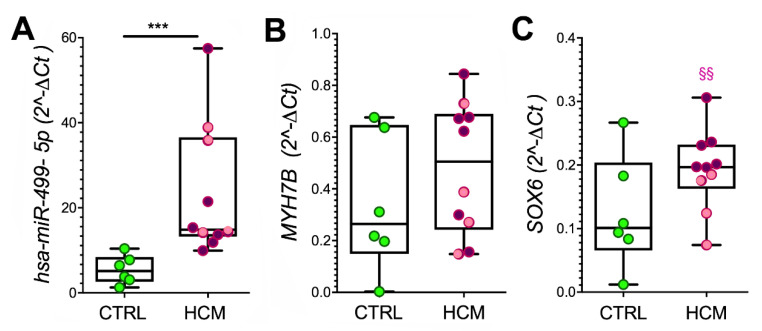
***MYH7B, miR-499*, and *SOX6* gene expression in cardiomyocytes.** The levels of expressed *miR-499* (**A**), *MYH7B* (**B**), and *SOX6* (**C**) are evaluated by RT-qPCR in cardiomyocyte samples isolated from a subset of myectomies using LCM. Values are presented as boxes (min to max), and dots indicate single sample values. The distribution of miR and gene levels in HCM patients without HCM-associated mutations are presented in violet; those bearing mutations are in pink. The *t*-test was applied, and significant differences between HCM and CTRL are shown as *** *p* < 0.001. Significant difference between HCM with or without mutation is evaluated by the Mann–Whitney test, §§ *p* < 0.01.

In addition, RT-qPCR showed higher expression of *miR-499* (*p* = 0.0005, Figure 3A), a trend towards increased expression of *MYH7B* and *SOX6* (Figure 3B,C), but comparable levels of *miR-208a* and *miR-208b* (Figure S7C) in the cardiomyocyte areas from HCM vs. CTRL. The expression level of *SOX6* was lower in samples from patients bearing mutations (3 patients with various mutations in *Myosin Binding Protein C3* and 1 patient with a mutation in both *Alpha-actinin-2* and *Myosin Light Chain Kinase 2*) than in those without mutations (*p* = 0.0095, Figure 3C). No further association with patient mutations was found.

### 3.7. MyomiRs in Plasma Samples

**Figure 4:**
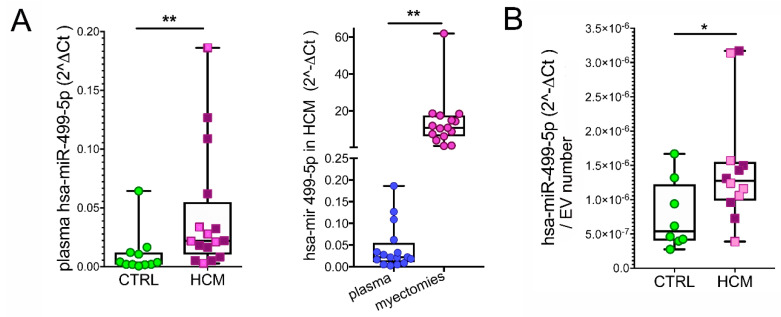
**Circulating *miR-499*.** The difference between hsa-miR-499-5p levels in plasma samples from HCM (n = 16) and healthy volunteers (CTRL, n = 11) is shown ((**A**)**, left plot**). The comparison between expression levels of hsa-miR-499-5p in myocardial and plasmatic samples from the same HCM patients is presented ((**A**)**, right plot**). The difference between hsa-miR-499-5p levels in plasma EV from subsets of HCM (n = 12) vs. CTRL (n = 8) is plotted (**B**). Mann–Whitney test and paired and unpaired *t*-tests are applied, respectively. *p*-value < 0.05 is considered significant and indicated by *; *p* < 0.01 is indicated by **.

To evaluate the potential of circulating MyomiRs in HCM, plasma samples from HCM patients and healthy volunteers were compared. The *miR-208a, miR-208b*, and *Mhrt* were below the detection limits of RT-qPCR in the plasma. However, *miR-499* was found in a significantly higher amount in HCM patients than in controls (*p* = 0.0017). Lower levels of *miR-499* were determined in plasma vs. myocardial samples, which may be partially justified by the dilution in the bloodstream (*p* = 0.0025, Figure 4A). The analysis of EV isolated from plasma indicated the *miR-499* as an exosomally carried miR and confirmed the above difference between HCM patients and healthy volunteers (*p* = 0.0495, Figure 4B).

### 3.8. Myosin and MyomiR Network in Cardiomyocytes from Human iPSC

To evaluate whether the above described features of the myosin and MyomiR network in patients with HCM treated with surgical myectomy may mirror regulatory feedback linking *miR-208a*, *MYH7*/*miR-208b/Mhrt*, *MYH7B*/*miR-499*, and *SOX6* [12], the cardiomyocytes derived from human dermal iPSC were transiently transfected with a mimic of either *miR-208a* or *miR-499* [46].

**Figure 5:**
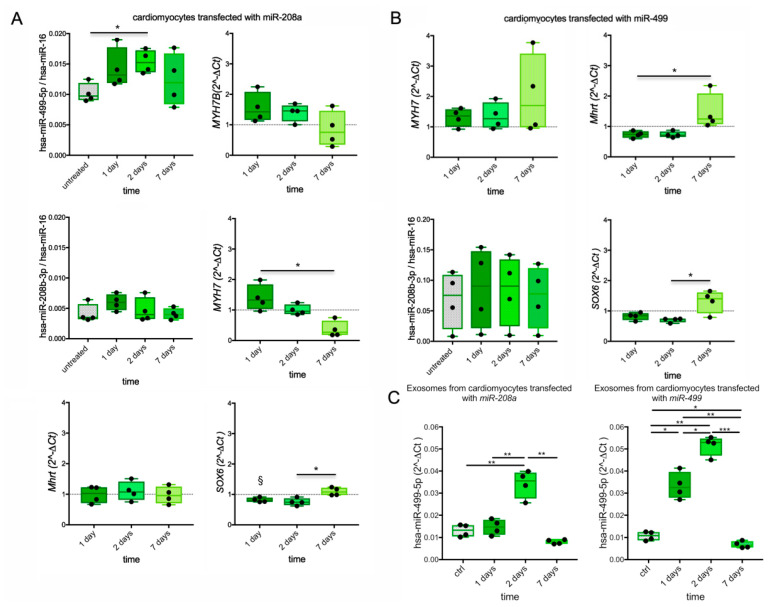
***MYHs*, MyomiRs, and *SOX6* expression in transfected cardiomyocytes from iPSC.** The levels of expressed *miR-499*, *MYH7B, MYH7, Mhrt, miR-208b*, and *SOX6* by RT-qPCR in cardiomyocytes differentiated from iPSC and transfected with *miR-208a* are shown at different time points (**A**). The expression of *MYH7, Mhrt, miR-208b*, and *SOX6* determined in cardiomyocytes after transfection with *miR-499* is plotted (**B**). The gene expression is presented as the ratio between 2^−ΔCt in treated vs. untreated cells (ratio control value =1 is indicated by dotted line where applicable). Amount of *miR-499* released from transfected cardiomyocytes into culture supernatants is also shown (**C**). Values are presented as boxes (min to max), and dots indicate single sample values. ANOVA and Friedman tests are applied. *p*-value < 0.05 is considered significant and shown as *; *p* < 0.01 is indicated by **, and *p* < 0.001 by ***. Significant difference (*p*-value < 0.05 ) vs. untreated cells is indicated by §.

On day 1 after transfection with *miR-208a* (Figure 5A), the levels of *miR-499* and *MYH7B,* but also of *MYH7*, increased (mean increase +40%, +40%, and +50%, respectively), and *SOX6* decreased (mean change −18%) in transfected vs. untreated cardiomyocytes. On day 2, *MYH7* diminished to a level comparable to that of untreated cells and decreased below it by day 7. Conversely, *miR-499* slightly but significantly increased (+50% *p* = 0.0428) on day 2, while *MYH7B* and *SOX6* remained stable both on day 1 and 2, but all recovered to untreated cell levels by day 7. Overall, this data showed a shorter effect of *miR-208a* on *MYH7* than on the other targets. The transfection with *miR-208a* did not lead to significant changes in *miR-208b* or *Mhrt.*

Following *miR-499* transfection, increases in *MYH7* (+31% at day 1, +35% at day 2) and decreases in *Mhrt* (−26% at both days 1 and 2) and *SOX6* (−18% at day 1, +30% at day 2) but no changes in *miR-208b* were detected. The effects of *miR-499* reverted to untreated cell levels or higher on day 7, when significant upregulation of *Mhrt* vs. day 1 (*p* = 0.0155) and *SOX6* vs. day 2 (*p* = 0.0370) were observed, suggesting a rebound effect.

EV-associated *miR-499* was detected in cell supernatants (Figure 5C and Figure S9). In *miR-208a*-transfected cardiomyocytes, the EV-associated *miR-499* transiently increased at day 2 (*p* = 0.0082 vs. CTRL, *p* = 0.0061 vs. day 1 and *p* = 0.0179 vs. day 7). In the *miR-499*-transfected cardiomyocytes, the EV-associated *miR-499* significantly increased from day 1 to day 2 (*p* = 0.0157 and *p* = 0.0007, respectively) before decreasing below the control level at day 7 (*p* = 0.0325).

## 4. Discussion

The present study demonstrates that the expression of the MyHC isoforms in the interventricular septum of patients with obstructive HCM is uncoupled from that of the encoding genes. The MyHC6 but not *MYH6* expression was reduced, and MyHC7 and MyHC7b did not increase, although the expression of both *MYH7* and *MYH7B* was higher in patients compared to CTRL.

We harnessed the power of MS proteomics as an affordable tool to investigate the link between HCM phenotypes and myosin isoforms [47,48], overcoming the potential bias related to antibody-based assays by identifying proteotypic peptides with unique occurrence in a single isoform. The observed reduction in the relative amount of MyHC6 (with no changes in MyLC2) is in line with previous studies on non-failing and failing human hearts with hypertrophic [49], ischemic, or dilated cardiomyopathy [50] (Table S3). The apparent discrepancy in SDS electrophoresis, long considered the gold standard for cardiac MyHC proteomics but never detecting MyHC7b, is possibly due to a different degree of sensitivity [5].

Overall, our data on *MYHs* confirm and expand on previous reports on αMyHC and βMyHC mRNA in human obstructive HCM [49] and failing hearts [3,5,6,51] (Table S3), supporting the occurrence of a switch from *MYH6* to *MYH7* and *MYH7B* at the nuclear level.

These results are indicative of an inefficient translation process involving *MYHs*, leading to MyHC levels comparable to controls for upregulated genes (i.e., *MYH7*/MyHC7: *MYH7B*/MyHC7b) but lower for non-deregulated genes (i.e., *MYH6*/MyHC6). Myoarchitectural disarray with heterogeneously-distributed hypertrophic cardiomyocytes is a hallmark of HCM myocardium [52] and may implicate the existence of local, scattered differences in the activity of *MYHs* aimed at producing MyHCs. In agreement, cell-to-cell, nonhomogeneous gene expression in cardiomyocytes has been proposed as a cause of abnormal activation of *MYH7* transcription [53]. Moreover, the low production of MyHC7b with respect to the encoding of *MYH7B* has been reported due to exon-skipping [54]. Our data did not exclude the production of truncated isoforms but show the presence of cardiac MyHC7b both in normal and HCM individuals, indicating that in vivo *MYH7B* is not as transcriptionally inactive as it can be in vitro [11].

The functional significance of limited pathological variation in myosins, in particular of those as poorly expressed as *MYH7B,* is still a matter of debate [49,51,54,55]. The explanation may reside in the non-coding transcripts generated by *MYHs*, i.e., *Mhrt* [9,10], *lncMYH7b* [11], and the MyomiRs (*miR-208a miR-208b* and *miR-499)* [9,10,12,56], which putatively regulate the function of numerous target genes (Table S3 and Supplementary Materials S2). Contrary to a report on a small cohort of individuals with LVH or ischemic or idiopathic dilated cardiomyopathy [10], in our study, the *Mhrt* expression significantly increased in HCM myectomy samples but not in plasma. Because *Mhrt* may play a protective epigenetic role in cardiac hypertrophy by controlling chromatin structure [10,57], our results suggest an attempt by the HCM heart to counteract hypertrophy.

Pathological status may also affect tissue and cell-specific stability as well as the amount of miRs [9,58]. Elevated circulating MyomiR levels have been associated with myocardial damage [59,60], and *miR-499* has recently been proposed as a circulating biomarker for HCM [61]. Association with carriers such as lipoproteins or EV increases miR stability, allowing the delivery of functional miRs to distant targets [62]; thus, the EV-association of *miR-499* in HCM plasma may support an extra-cardiac role.

A functional interplay among myosins, MyomiRs, and *SOX6* (target gene of *miR-499*) was proposed in animal models. Myocardial elevation of *miR-499* without significant alteration of *SOX6* has been quantitatively associated with the up-regulation of the β*MyHC* gene, cardiac hypertrophy, and an increase in cardiomyocyte size, but also with genes associated with stress-induced cardiac dysfunction in mice [13]. Moreover, the inactivation of *miR-208a* hampered the induction of the *βMyHC* gene in mice with cardiac hypertrophy [9] and was associated with increased *SOX6* in those with cardiac hyperaldosteronism [63]. Conversely, in HCM myocardium, the upregulation of *MYH7*, *MYH7B*, and *miR-499* was paralleled by upregulated *SOX6,* which was increased not only in cardiomyocytes but also in coronary arterioles-containing areas. The opposite reciprocal expression of *MYH7* and *MYH6* in rodent and human ventricles and the genetic heterogeneity in patients complicate the comparison with animal models.

Overall, this snapshot of the myosin/MyomiR network in obstructive HCM is novel but lacks an underlying mechanism. To shed some light on the mechanism, we used human iPSC-derived cardiomyocytes to investigate the feedback circuit functionally linking MyomiRs, *MYHs*, and *SOX6* in skeletal muscle [12]. Cardiomyocyte transfection demonstrated mild modulatory efficacy of *miR-208a* and *miR-499* at early stages (days 1 and 2), but a rebound effect at a late stage (within day 7) on *MYH7*, *Mhrt*, and *SOX6* in *miR-499*-transfected cells and on *SOX6* in *miR-208a*-transfected cells. A two-step process could be envisaged (Figure S8), and we hypothesize that the snapshot of the myosin/MyomiR network in the HCM cohort partially reflects step 2. Because in the HCM myocardium, the miRs are not fully lost (and *miR-499* is partially released into the bloodstream), their regulatory effects might persist (e.g., the moderate upregulation of *MYH7b*), an effect not seen in transiently transfected cells. The scant changes in *MYH7* and *MYH7B* following in vitro overexpression of *miR-208a* are in agreement with the finding that *MYH6* (and consequently *miR-208a*) was not increased in the human HCM myocardium; thus, a secondary role for *miR-208a* may be conceivable in obstructive HCM. The uncoupling between genes and proteins indicates that *MYHs* are not fully transcriptionally active, leading to a global defect in MyHCs and ultimately to altered sarcomeric contractility, completing this picture.

Put in perspective, in vitro studies investigating the possible antagonism/synergy between MyomiRs on *SOX6* and the interaction between myosins and MyomiRs on cardiomyocytes submitted to mechanical stretch could further improve our knowledge of the molecular mechanism in HCM patients. However, this is beyond the purpose of our study. Myosin modulation is the object of randomized clinical trials (e.g., the successfully completed Explorer HCM phase 3 study with mavacamten and the ongoing Sequoia HCM phase 3 study with aficamten). Consequently, differences in myosins associated with an altered MyomiR network may represent not only a relevant pathophysiologic trait in HCM but also a potential molecular target for mavacamten, which could induce a relative normalization of the myosin/MyomiR network in treated patients.

## 5. Limitations

We acknowledge several limitations in this proof-of-concept study. First, it is impossible to ascertain from these findings whether the observed changes are specific to obstructive HCM or may also play a role in patients with other cardiomyopathies or secondary LVH. Whether myosin/MyomiR network abnormalities are also present in such conditions remains unknown. Ex vivo investigation of the human myocardium provides a snapshot of the tissue condition at the time of collection. Thus, we cannot rule out that differences in tissue processing between patients and controls might have, at least in part, affected the results. This is an unavoidable limitation shared by similar previous studies.

Some of these samples had already been used in the frame of the same research project [41,64], and few of them did not suffice to be used in all the assays. The limited quantity of plasma donated by HCM patients and of transfected iPSC supernatants allowed for the basic characterization of EV and the determination of the *miR-499* associated with EV but did not discriminate between miRs wrapped inside vesicles and bound to the EV surface.

Lastly, our study focused on quantitative protein/gene defects, which can only partially explain abnormal sarcomeric function in HCM. The latter likely involves a spectrum of post-translational modifications, ranging from altered phosphorylation of MyLC2 [65] to three-dimensional structure changes, such as variation in the spatial orientation of myosin heads [66], highlighting the complexity in contractility defects of the HCM myocardium.

## 6. Conclusions

In patients with obstructive HCM, we found an uncoupling between genes and MyHC and associated changes in myosin heavy chains and the MyomiR network, supporting a modulatory function of MyomiRs on the HCM phenotype, possibly contributing to phenotypic diversity and providing putative therapeutic targets.

## Figures and Tables

**Table 1:** Baseline characteristics of HCM patients.

Demographic Data	N = 23
Age (years), M (SD)	59 (9)
Gender (male), N (%)	13 (56.5)
BMI (Kg/m^2^), M (SD)	27.9 (5.1)
Creatinine (mg/dL), M (SD)	0.84 (0.33)
GFR (mL/min)	110.9 (44.8)
Troponin T (ng/L) M (SD)	545.7 (461)
**Clinical data**	
Family history of HCM, N (%)	6 (26)
Family history of SCD, N (%)	4 (17)
NYHA ≥ III, N (%)	9 (39)
Angina, N (%)	6 (26)
Syncope, N (%)	6 (26)
Atrial fibrillation (previous history), N (%)	4 (17)
NSVT, N (%)	1 (4)
ICD, N (%)	0
**Medical Therapy**	
Beta-blockers, N (%)	21 (91)
Antiarrhythmic drugs, N (%)	5 (21)
Diuretics, N (%)	18 (74)
RAAS-i, N (%)	3 (13)

**Legend:** M, mean; SD, standard deviation; N, number; %, percentage; NYHA, New York Heart Association; SCD, sudden cardiac death; HCM, hypertrophic cardiomyopathy; ICD, implantable cardioverter defibrillator; NSVT, non-sustained ventricular tachycardia; RAAS-i, renin-angiotensin-aldosterone system inhibitor.

**Table 2:** Echocardiographic data of HCM patients.

Echocardiographic Structural Data.	
IVS thickness (mm), M (SD)	21 (5.6)
PW thickness (mm), M (SD)	12 (4)
IVS/PW ratio, M (SD)	1.99 (0.88)
LV-EDD (mm), M (SD)	43.6 (7.3)
BSA (m^2^), M (SD)	1.92 (0.26)
LA volume indexed, M (SD)	45.90 (18.0)
**Systolic function**	
LV-EF (%), M (SD)	67 (9)
TDI s’ peak (cm/s), M (SD)	9 (2)
**Diastolic function**	
E peak (cm/s), M (SD)	64 (27)
A peak (cm/s), M (SD)	78 (37)
E/A ratio, M (SD)	1.0 (0.83)
DT (ms), M (SD)	184 (84)
IVRT (ms), M (SD)	78 (20)
TDI e’ peak (cm/s), M (SD)	9 (2)
TDI, a’ peak (cm/s), M (SD)	12 (4)
E/e’ ratio	8 (5)
PAPs (mmHg), M (SD)	31 (7)
Mitral regurgitation ≥ grade 3, N (%)	8 (35)
SAM-related LVOT-max gradient at rest (mmHg), M (SD)	80 (29)

**Legend:** M, mean; SD, standard deviation; N, number; %, percentage; IVS, interventricular septum; PW, posterior wall; LV, left ventricular; EDD, end-diastolic diameter; LA, left atrial; BSA, body surface area; EF, ejection fraction; TDI, tissue Doppler imaging; E, early filling velocity; A, atrial contraction velocity; DT, deceleration time; IVRT, iso-volumetric relaxation time; SAM, aystolic anterior motion; LVOT, left ventricular outflow tract.

## Data Availability

The main data presented in this study are available either in the article Tables or in Supplementary Materials. Single patient data are subject to privacy law and available on request from the corresponding author.

## Supplementary Materials

The following supporting information can be downloaded at: https://www.mdpi.com/article/10.3390/biomedicines10092180/s1: Supplementary Materials S1, Supplementary Materials S2.

## References

[1] Olivotto I., Cecchi F., Poggesi C., Yacoub M.H. (2012). Patterns of disease progression in hypertrophic cardiomyopathy: An individualized approach to clinical staging. Circ. Heart Fail..

[2] Marian A.J. (2021). Molecular Genetic Basis of Hypertrophic Cardiomyopathy. Circ. Res..

[3] Lowes B.D., Minobe W., Abraham W.T., Rizeq M.N., Bohlmeyer T.J., Quaife R.A., Roden R.L., Dutcher D.L., Robertson A.D., Voelkel N.F. (1997). Changes in gene expression in the intact human heart. Downregulation of alpha-myosin heavy chain in hypertrophied, failing ventricular myocardium. J. Clin. Investig..

[4] Morano I., Hadicke K., Grom S., Koch A., Schwinger R.H., Bohm M., Bartel S., Erdmann E., Krause E.G. (1994). Titin, myosin light chains and C-protein in the developing and failing human heart. J. Mol. Cell Cardiol..

[5] Miyata S., Minobe W., Bristow M.R., Leinwand L.A. (2000). Myosin heavy chain isoform expression in the failing and nonfailing human heart. Circ. Res..

[6] Nakao K., Minobe W., Roden R., Bristow M.R., Leinwand L.A. (1997). Myosin heavy chain gene expression in human heart failure. J. Clin. Investig..

[7] Peter A.K., Rossi A.C., Buvoli M., Ozeroff C.D., Crocini C., Perry A.R., Buvoli A.E., Lee L.A., Leinwand L.A. (2019). Expression of Normally Repressed Myosin Heavy Chain 7b in the Mammalian Heart Induces Dilated Cardiomyopathy. J. Am. Heart Assoc..

[8] Reiser P.J. (2019). Current understanding of conventional and novel co-expression patterns of mammalian sarcomeric myosin heavy chains and light chains. Arch. Biochem. Biophys..

[9] van Rooij E., Sutherland L.B., Qi X., Richardson J.A., Hill J., Olson E.N. (2007). Control of stress-dependent cardiac growth and gene expression by a microRNA. Science.

[10] Han P., Li W., Lin C.H., Yang J., Shang C., Nuernberg S.T., Jin K.K., Xu W., Lin C.Y., Lin C.J. (2014). A long noncoding RNA protects the heart from pathological hypertrophy. Nature.

[11] Broadwell L.J., Smallegan M.J., Rigby K.M., Navarro-Arriola J.S., Montgomery R.L., Rinn J.L., Leinwand L.A. (2021). Myosin 7b is a regulatory long noncoding RNA (lncMYH7b) in the human heart. J. Biol. Chem..

[12] van Rooij E., Quiat D., Johnson B.A., Sutherland L.B., Qi X., Richardson J.A., Kelm R.J., Olson E.N. (2009). A family of microRNAs encoded by myosin genes governs myosin expression and muscle performance. Dev. Cell.

[13] Shieh J.T., Huang Y., Gilmore J., Srivastava D. (2011). Elevated miR-499 levels blunt the cardiac stress response. PLoS ONE.

[14] Sluijter J.P., van Mil A., van Vliet P., Metz C.H., Liu J., Doevendans P.A., Goumans M.J. (2010). MicroRNA-1 and -499 regulate differentiation and proliferation in human-derived cardiomyocyte progenitor cells. Arter. Thromb. Vasc. Biol..

[15] van Rooij E., Sutherland L.B., Liu N., Williams A.H., McAnally J., Gerard R.D., Richardson J.A., Olson E.N. (2006). A signature pattern of stress-responsive microRNAs that can evoke cardiac hypertrophy and heart failure. Proc. Natl. Acad. Sci. USA.

[16] Saleem M., Rahman S., Elijovich F., Laffer C.L., Ertuglu L.A., Masenga S.K., Kirabo A. (2022). Sox6, A Potential Target for MicroRNAs in Cardiometabolic Disease. Curr. Hypertens. Rep..

[17] Nachtigall P.G., Dias M.C., Carvalho R.F., Martins C., Pinhal D. (2015). MicroRNA-499 expression distinctively correlates to target genes sox6 and rod1 profiles to resolve the skeletal muscle phenotype in Nile tilapia. PLoS ONE.

[18] de Oliveira Silva T., Lino C.A., Miranda J.B., Balbino-Silva C.S., Lunardon G., Lima V.M., Jensen L., Donato J., Irigoyen M.C., Barreto-Chaves M.L.M. (2022). The miRNA-143–3p-Sox6-Myh7 pathway is altered in obesogenic diet-induced cardiac hypertrophy. Exp. Physiol..

[19] Bell M.L., Buvoli M., Leinwand L.A. (2010). Uncoupling of expression of an intronic microRNA and its myosin host gene by exon skipping. Mol. Cell Biol..

[20] McCarthy J.J., Esser K.A., Peterson C.A., Dupont-Versteegden E.E. (2009). Evidence of MyomiR network regulation of beta-myosin heavy chain gene expression during skeletal muscle atrophy. Physiol. Genom..

[21] Satoh M., Minami Y., Takahashi Y., Tabuchi T., Nakamura M. (2010). Expression of microRNA-208 is Associated with Adverse Clinical Outcomes in Human Dilated Cardiomyopathy. J. Card. Fail..

[22] Prodanovic M., Geeves M.A., Poggesi C., Regnier M., Mijailovich S.M. (2022). Effect of Myosin Isoforms on Cardiac Muscle Twitch of Mice, Rats and Humans. Int. J. Mol. Sci..

[23] Elliott P.M., Anastasakis A., Borger M.A., Borggrefe M., Cecchi F., Charron P., Hagege A.A., Lafont A., Limongelli G., Mahrholdt H. (2014). 2014 ESC Guidelines on diagnosis and management of hypertrophic cardiomyopathy: The Task Force for the Diagnosis and Management of Hypertrophic Cardiomyopathy of the European Society of Cardiology (ESC). Eur. Heart J..

[24] Richards S., Aziz N., Bale S., Bick D., Das S., Gastier-Foster J., Grody W.W., Hegde M., Lyon E., Spector E. (2015). Standards and guidelines for the interpretation of sequence variants: A joint consensus recommendation of the American College of Medical Genetics and Genomics and the Association for Molecular Pathology. Genet. Med..

[25] Li Q., Wang K. (2017). InterVar: Clinical Interpretation of Genetic Variants by the 2015 ACMG-AMP Guidelines. Am. J. Hum. Genet..

[26] Whiffin N., Walsh R., Govind R., Edwards M., Ahmad M., Zhang X., Tayal U., Buchan R., Midwinter W., Wilk A.E. (2018). CardioClassifier: Disease- and gene-specific computational decision support for clinical genome interpretation. Genet. Med..

[27] Agbulut O., Li Z., Mouly V., Butler-Browne G.S. (1996). Analysis of skeletal and cardiac muscle from desmin knock-out and normal mice by high resolution separation of myosin heavy-chain isoforms. Biol. Cell.

[28] Arnostova P., Jedelsky P.L., Soukup T., Zurmanova J. (2011). Electrophoretic mobility of cardiac myosin heavy chain isoforms revisited: Application of MALDI TOF/TOF analysis. J. Biomed. Biotechnol..

[29] Shevchenko A., Wilm M., Vorm O., Mann M. (1996). Mass spectrometric sequencing of proteins silver-stained polyacrylamide gels. Anal. Chem..

[30] Rauniyar N. (2015). Parallel Reaction Monitoring: A Targeted Experiment Performed Using High Resolution and High Mass Accuracy Mass Spectrometry. Int. J. Mol. Sci..

[31] Schwenk J.M., Omenn G.S., Sun Z., Campbell D.S., Baker M.S., Overall C.M., Aebersold R., Moritz R.L., Deutsch E.W. (2017). The Human Plasma Proteome Draft of 2017: Building on the Human Plasma PeptideAtlas from Mass Spectrometry and Complementary Assays. J. Proteome Res..

[32] MacLean B., Tomazela D.M., Shulman N., Chambers M., Finney G.L., Frewen B., Kern R., Tabb D.L., Liebler D.C., MacCoss M.J. (2010). Skyline: An open source document editor for creating and analyzing targeted proteomics experiments. Bioinformatics.

[33] Stuart C.A., Stone W.L., Howell M.E., Brannon M.F., Hall H.K., Gibson A.L., Stone M.H. (2016). Myosin content of individual human muscle fibers isolated by laser capture microdissection. Am. J. Physiol. Cell Physiol..

[34] Pistilli D., di Gioia C.R., D’Amati G., Sciacchitano S., Quaglione R., Quitadamo R., Casali C., Gallo P., Santorelli F.M. (2003). Detection of deleted mitochondrial DNA in Kearns-Sayre syndrome using laser capture microdissection. Hum. Pathol..

[35] Pianezzi E., Altomare C., Bolis S., Balbi C., Torre T., Rinaldi A., Camici G.G., Barile L., Vassalli G. (2020). Role of somatic cell sources in the maturation degree of human induced pluripotent stem cell-derived cardiomyocytes. BBA-Mol. Cell Res..

[36] Balbi C., Milano G., Fertig T.E., Lazzarini E., Bolis S., Taniyama Y., Sanada F., Di Silvestre D., Mauri P., Gherghiceanu M. (2021). An exosomal-carried short periostin isoform induces cardiomyocyte proliferation. Theranostics.

[37] Burrello J., Biemmi V., Dei Cas M., Amongero M., Bolis S., Lazzarini E., Bollini S., Vassalli G., Paroni R., Barile L. (2020). Sphingolipid composition of circulating extracellular vesicles after myocardial ischemia. Sci. Rep. UK.

[38] Larios J., Mercier V., Roux A., Gruenberg J. (2020). ALIX- and ESCRT-III-dependent sorting of tetraspanins to exosomes. J. Cell Biol..

[39] Willms E., Johansson H.J., Mager I., Lee Y., Blomberg K.E.M., Sadik M., Alaarg A., Smith C.I.E., Lehtio J., Andaloussi S.E.L. (2016). Cells release subpopulations of exosomes with distinct molecular and biological properties. Sci. Rep. UK.

[40] Lee S.S., Won J.H., Lim G.J., Han J., Lee J.Y., Cho K.O., Bae Y.K. (2019). A novel population of extracellular vesicles smaller than exosomes promotes cell proliferation. Cell Commun. Signal..

[41] Lombardi M., Lazzeroni D., Benedetti G., Bertoli G., Lazarevic D., Riba M., De Cobelli F., Rimoldi O., d’Amati G., Olivotto I. (2021). Plasmatic and myocardial microRNA profiles in patients with Hypertrophic Cardiomyopathy. Clin. Transl. Med..

[42] Livak K.J., Schmittgen T.D. (2001). Analysis of relative gene expression data using real-time quantitative PCR and the 2(-Delta Delta C(T)) Method. Methods.

[43] Dobin A., Davis C.A., Schlesinger F., Drenkow J., Zaleski C., Jha S., Batut P., Chaisson M., Gingeras T.R. (2013). STAR: Ultrafast universal RNA-seq aligner. Bioinformatics.

[44] Chen Y., Wang X. (2020). miRDB: An online database for prediction of functional microRNA targets. Nucleic Acids Res..

[45] Szklarczyk D., Gable A.L., Lyon D., Junge A., Wyder S., Huerta-Cepas J., Simonovic M., Doncheva N.T., Morris J.H., Bork P. (2019). STRING v11: Protein-protein association networks with increased coverage, supporting functional discovery in genome-wide experimental datasets. Nucleic Acids Res..

[46] Barile L., Lionetti V., Cervio E., Matteucci M., Gherghiceanu M., Popescu L.M., Torre T., Siclari F., Moccetti T., Vassalli G. (2014). Extracellular vesicles from human cardiac progenitor cells inhibit cardiomyocyte apoptosis and improve cardiac function after myocardial infarction. Cardiovasc. Res..

[47] Coats C.J., Heywood W.E., Virasami A., Ashrafi N., Syrris P., Dos Remedios C., Treibel T.A., Moon J.C., Lopes L.R., McGregor C.G.A. (2018). Proteomic Analysis of the Myocardium in Hypertrophic Obstructive Cardiomyopathy. Circ. Genom. Precis. Med..

[48] Helmke S.M., Yen C.Y., Cios K.J., Nunley K., Bristow M.R., Duncan M.W., Perryman M.B. (2004). Simultaneous quantification of human cardiac alpha- and beta-myosin heavy chain proteins by MALDI-TOF mass spectrometry. Anal. Chem..

[49] Ritter O., Luther H.P., Haase H., Baltas L.G., Baumann G., Schulte H.D., Morano I. (1999). Expression of atrial myosin light chains but not alpha-myosin heavy chains is correlated in vivo with increased ventricular function in patients with hypertrophic obstructive cardiomyopathy. J. Mol. Med..

[50] Reiser P.J., Moravec C.S. (2014). Sex differences in myosin heavy chain isoforms of human failing and nonfailing atria. Am. J. Physiol. Heart Circ. Physiol..

[51] Abraham W.T., Gilbert E.M., Lowes B.D., Minobe W.A., Larrabee P., Roden R.L., Dutcher D., Sederberg J., Lindenfeld J.A., Wolfel E.E. (2002). Coordinate changes in Myosin heavy chain isoform gene expression are selectively associated with alterations in dilated cardiomyopathy phenotype. Mol. Med..

[52] Marian A.J., Braunwald E. (2017). Hypertrophic Cardiomyopathy Genetics, Pathogenesis, Clinical Manifestations, Diagnosis, and Therapy. Circ. Res..

[53] Kraft T., Montag J., Radocaj A., Brenner B. (2016). Hypertrophic Cardiomyopathy: Cell-to-Cell Imbalance in Gene Expression and Contraction Force as Trigger for Disease Phenotype Development. Circ. Res..

[54] Lee L.A., Broadwell L.J., Buvoli M., Leinwand L.A. (2021). Nonproductive Splicing Prevents Expression of MYH7b Protein in the Mammalian Heart. J. Am. Heart Assoc..

[55] Lee L.A., Karabina A., Broadwell L.J., Leinwand L.A. (2019). The ancient sarcomeric myosins found in specialized muscles. Skelet. Muscle.

[56] Matkovich S.J., Hu Y., Eschenbacher W.H., Dorn L.E., Dorn G.W. (2012). Direct and indirect involvement of microRNA-499 in clinical and experimental cardiomyopathy. Circ. Res..

[57] Liu J., Wang D.Z. (2014). An epigenetic “LINK(RNA)” to pathological cardiac hypertrophy. Cell Metab..

[58] Kingston E.R., Bartel D.P. (2019). Global analyses of the dynamics of mammalian microRNA metabolism. Genome Res..

[59] Corsten M.F., Dennert R., Jochems S., Kuznetsova T., Devaux Y., Hofstra L., Wagner D.R., Staessen J.A., Heymans S., Schroen B. (2010). Circulating MicroRNA-208b and MicroRNA-499 reflect myocardial damage in cardiovascular disease. Circ. Cardiovasc. Genet..

[60] Wang J., Xu L., Tian L., Sun Q. (2021). Circulating microRNA-208 family as early diagnostic biomarkers for acute myocardial infarction: A meta-analysis. Medicine.

[61] Baulina N., Pisklova M., Kiselev I., Chumakova O., Zateyshchikov D., Favorova O. (2022). Circulating miR-499a-5p Is a Potential Biomarker of MYH7-Associated Hypertrophic Cardiomyopathy. Int. J. Mol. Sci..

[62] Min P.K., Chan S.Y. (2015). The biology of circulating microRNAs in cardiovascular disease. Eur. J. Clin. Investig..

[63] Azibani F., Devaux Y., Coutance G., Schlossarek S., Polidano E., Fazal L., Merval R., Carrier L., Solal A.C., Chatziantoniou C. (2012). Aldosterone inhibits the fetal program and increases hypertrophy in the heart of hypertensive mice. PLoS ONE.

[64] Lombardi M., Lazzeroni D., Pisano A., Girolami F., Alfieri O., La Canna G., d’Amati G., Olivotto I., Rimoldi O.E., Foglieni C. (2020). Mitochondrial Energetics and Ca^2+^-Activated ATPase in Obstructive Hypertrophic Cardiomyopathy. J. Clin. Med..

[65] Claes G.R., van Tienen F.H., Lindsey P., Krapels I.P., Helderman-van den Enden A.T., Hoos M.B., Barrois Y.E., Janssen J.W., Paulussen A.D., Sels J.W. (2016). Hypertrophic remodelling in cardiac regulatory myosin light chain (MYL2) founder mutation carriers. Eur. Heart J..

[66] Trivedi D.V., Adhikari A.S., Sarkar S.S., Ruppel K.M., Spudich J.A. (2018). Hypertrophic cardiomyopathy and the myosin mesa: Viewing an old disease in a new light. Biophys. Rev..

