# Supplementary material for: Myosins and MyomiR Network in Patients with Obstructive Hypertrophic Cardiomyopathy
Source: Biomedicines. 2022 Sep 3;10(9):2180. doi: 10.3390/biomedicines10092180 (PMC9496008; doi:10.3390/biomedicines10092180)
Supplement: Supplementary file 1 [file biomedicines-10-02180-s001.zip › Supplemental Material_Foglieni C_Biomedicines_SI.pdf]

Supplemental material to:

Myosins and MyomiR network in patients with obstructive hypertrophic cardiomyopathy

Chiara Foglieni, Maria Lombardi, Davide Lazzeroni, Riccardo Zerboni, Edoardo Lazzarini, Gloria Bertoli, Annalinda Pisano, Francesca Girolami, Annapaola Andolfo, Cinzia Magagnotti, Giovanni Peretto, Carmem L. Sartorio, Iacopo Olivotto, Giovanni La Canna, Ottavio Alfieri, Ornella E. Rimoldi, Lucio Barile, Giulia d'Amati, Paolo G. Camici

Table S1. Genes analyzed in the study.

**Gene**	**Transcript**
ACTC1	NM_005159.4
ACTN2	NM_001103.2
CALR3	NM_145046.3
CAV3	NM_033337.2
CSRP3	NM_003476.2
GLA	NM_000169.2
JPH2	NM_020433.4
LAMP2	NM_002294.2
MYBPC3	NM_000256.3
MYH6	NM_004999.3
MYH7	NM_000257.2
MYL2	NM_000432.3
MYL3	NM_000258.2
MYLK2	NM_033118.3
MYOZ2	NM_016599.3
MYPN	NM_032578.2
NEXN	NM_144573.3
PLN	NM_002667.3
PRKAG2	NM_016203.3
TCAP	NM_003673.3
TNNC1	NM_003280.1
TNNI3	NM_000363.4
TNNT2	NM_001001430
TPM1	NM_000366.5
TTR	NM_000371.3
VCL	NM_014000.2

Table S2. Variants identified in HCM patients

patient ID	Gene	Sequence Variant Nomenclature (HGVS) *	Variant		Publication on Human Gene Mutation Database **	Sequence Variant Classification (ACMG)***
			protein	type		
**1**	negative					
**2**	negative					
**3**	negative					
**4**	negative					
**5**	MYBPC3	c.1458-1G>A	p.(?)	splice site	Walsh et al. Genet. Med. 2017 doi:10.1038/gim.2016.90.	Class 5-Pathogenic
	MYBPC3	c.649A>G	p.Ser217Gly	missense	Viswanathan et al. PLoS ONE 2017 doi:10.1371/journal.pone.0187948	Class 3-Unknown pathogenicity
	CAV3	c.233C>T	p.Thr78Met	missense	Vatta et al. Circulation 2006 DOI:10.1161/CIRCULATIONAHA.106.635268	Class 3-Unknown pathogenicity
**6**	MYBPC3	c.772G>A	p.Glu258Lys	missense / splice site	Niimura et al. NEJM 1998 DOI:10.1056/NEJM199804303381802	Class 5-Pathogenic
**7**	MYBPC3	c.3767_3769delCCA	p.Thr1256del	inframe del	Coppini et al. JACC 2014 doi:10.1016/j.jacc.2014.09.059	Class 4-Likely pathogenic
**8**	negative					
**9**	negative					
**10**	negative					
**11**	ACTN2	c.1930 G>A	p.Ala644Thr	missense		Class 3-Unknown pathogenicity
	MYLK2	c.1768 G>T	p.Ala590Ser)	missense		Class 3-Unknown pathogenicity

**12**	negative					
**13**	MYPN	c.3335C>T	p.Pro1112Leu	missense	Bagnall et al. IJC 2010 doi:10.1016/j.ijcard.2010.08.004	Class 3-Unknown pathogenicity
	negative					
**15**	negative					
**16**	negative					
**17**	MYH7	c.428G>A	p.Arg143Gln	missense	Homburger et al. PNAS 2016 doi:10.1073/pnas.1606950113	Class 4-Likely pathogenic
	MYPN	c.3335C>T	p.Pro1112Leu	missense	Bagnall et al. IJC 2010 doi:10.1016/j.ijcard.2010.08.004	Class 3-Unknown pathogenicity
**18**	negative					
**19**	negative					
**20**	negative					
**21**	MYBPC3	c.3192dupC	p.Lys1065Glnfs*12	frame shift	Girolami et al. JACC 2010 doi: 10.1016/j.jacc.2009.11.062	Class 5-Pathogenic
	MYBPC3	c.1112C>G	p.Pro371Arg	missense	Girolami et al. JACC 2010 doi: 10.1016/j.jacc.2009.11.062	Class 3-Unknown pathogenicity
	MYBPC3	c.1321G>A	p.Glu441Lys	missense	Olivotto et al. Mayo Clin Proc. 2008 doi: 10.4065/83.6.630	Class 3-Unknown pathogenicity
	MYBPC3	c.2908C>T	p.Arg970Trp	missense	Berge et al. Clin. Genet. 2014 doi: 10.1111/cge.12286	Class 4-Likely pathogenic
**22**	MYH7	c.3900G>C	p.Gln1300His	missense		Class 3-Unknown pathogenicity
**23**	MYH7	c.976G>C	p.Ala326Pro	missense	Michels et al. EHJ 2009 doi:10.1093/eurheartj/ehp306	Class 3-Unknown pathogenicity

Legend: * Sequence Variant Nomenclature based on Human Genome Variation Society nomenclature (**HGVS**)
Human Gene Mutation Database (HGMD®**) represents an attempt to collate all known (published) gene lesions responsible for human inherited disease.
*** Sequence Variant Classification based on American College of Medical Genetics and Genomics (**ACMG**) guidelines.

Table S3. Myosin and MyomiR changes in humans - a synopsis of selected published data.

	HCM	DCM	LVH and HF	DOI	PMID	citation
β -myosin y chain s (MYH)		DCM with HF vs. donor hearts; n unknown. PCR: no changes in β-MYH in myectomies		10.1161/01.CIR.83.6.1866	**2040039**	Feldman AM et al. Circulation. 1991
		DCM with HF(n=6) vs. donor hearts (n=6). RT-PCR: decrease in α-MYH and increase in β-MYH genes in LV myectomies		10.1172/JCI119770	**9410910**	Lowes BD et al. J Clin Invest. 1997
			Chronic HF (n=19) vs. candidate donors (n=7 non failing, n=7 heart dysfunction). PCR: α-MYH decreased in HF	10.1172/JCI119776	**9410916**	Nakao et al. J Clin Invest. 1997
		DCM with HF (n=47) treated with: Placebo/Metoprolol/Carvedilol. Effect: both α - and β -MYH genes incrementally increased in myectomies with the function change groups.			**12520092**	Abraham WT et al. *Mol Med*. 2002
β -myosin y chain ;) protein		DCM (n=12), ischemic cardiomyopathy (n=12) vs. heart donors (n=14), human fetal heart (n=12: atrial and ventricular samples). Electrophoresis: β-MyHC predominant in fetal & non failing ventricles < failing; the opposite for α-MyHCs in atria		10.1152/ajpheart.2001.280.4.H1814	**11247796**	Reiser PJ et al. Am J Physiol Heart Circ Physiol. 2001
			End-stage HF (n=40) and non failing (n=37) atria_ gender matched. Electrophoresis β-MyHC>α-MyHC independently from gender, β-MyHC gender -related differences left vs. right atria	0.1152/ajpheart.00810.2013	**24878771**	Reiser PJ , Moravec CS Am J Physiol Heart Circ Physiol. 2014
	HCM with LVOT (n=51), controls (n=7) and aortic stenosis (n=7). Proteomics by label-free Mass Spectrometry in myectomies: MyHC6, MyHC7, MyHC7B found in the pilot phase; MyHC7 reported in validation without intergroup differences			10.1161/CIRCGEN.117.001974	**30562113**	Coats CJ et al. Circ Genom Precis Med. 2018
2 protein			End-stage HF vs. adult normal, human embryonic heart. Electrophoresis: no changes in total MyHC, low MYL2 in embryonic samples	10.1006/jmcc.1994.1045	**8028019**	Morano I et al. J Mol Cell Cardiol 1994
			Chronic HF (n=16), healthy controls (n=12). IHC and WB in right auricle: MyLC2 was down-expressed in HF tissues	10.1002/clc.20832	**21259275**	Li Y etal. Clin Cardiol. 2011
β -myosin y chain MYH) and n (MyHC), yLC2		HF (DCM n=7, CAD n=3) vs. organ donors (n=12). Electrophoresis: decreased α-MYH and α-MyHC in HF		10.1161/01.RES.86.4.386	**10700442**	Miyata, S et al. Circ Res. 2000
	obstructive HCM (n=10) vs. donor hearts (n=4)_ interventricular septum. qualitative PCR & electrophoresis: β-MYH α-MYH mRNA, in HCM expressed MyLC2, not α-MyH			10.1007/s001099900030	**10569205**	Ritter O et al. J Mol Med (Berl). 1999
Mhrt		DCM (n=6) vs. LVH(n=5) vs.ischemic cardiomyopathy (n=3) vs. ctrl (n=4). RT-qPCR decrease in Mhrt in cardiovascular disease vs. ctrl myectomies		0.1038/nature13596	**25119045**	Han P et al. *Nature*. 2014
omiRs		DCM (n=6) vs. normal hearts (n=6). Northern blot: correlation of α-MYH with pre-miR-208 but not mature miR-208		10.1126/science.1139089	**17379774**	*van Rooij E et al. Science*. 2007
			AMI (n=33) vs. non-AMI patients with chest distress and pain (n= 33, i.e. 16 with CAD and 17 patients with other CVD) vs. healthy subjects (n=4). RT-qPCR in plasma: no miR-208a, low miR-499 in healthy people; increased miR-208a, low miR-499 in AMI only	10.1093/eurheartj/ehq013	**20159880**	Wang GK et al. Eur Heart J. 2010
			HF (n=15, non failing hearts (n=5), and hypertrophied non-failing hearts (n=2). MicroRNA arrays: increased miR-208, miR-499 and myh7b gene in HF	10.1161/CIRCRESAHA.112.265736	**22752967**	Matkovich SJ et al. Circ Res. 2012
			AF (n=4) vs. sinus rhythm (n=4) patients. Microarrays: miR-499 upregulated in AF	10.1016/j.hrthm.2013.03.005	**23499625**	Ling TY et al. Heart Rhythm. 2013
			Essential hypertension with LVH (n=50) vs. healthy individuals (n=30). RT-qPCR in peripheral blood samples increased miR-208 in cardiac hypertrophy	10.3892/etm.2015.2645	**26622415**	Huang X et al. *Exp Ther Med*. 2015
			AMI (n=142) non-AMI (n= 85) patients vs. normal subjects (n=100). Plasma miR-499 AMI > non AMI = normal; miR-499 increased overtime with chest pain in AMI, in relation with CK_MB and cTnI	10.3978/j.issn.2072-1439.2015.02.05	**:25922707**	Zhang L et al. J Thorac Dis. 2015
; -myosin y chain (MYH) & omiRs		DCM (n=82) vs. subjects without LV dysfunction (n=21). RT-qPCR: decreased α-MYH, increased β-MYH , miR-208, miR-208b, and miR-499 in DCM		10.1016/j.cardfail.2010.01.002	**20447577**	Satoh M et al.Journal of cardiac failure 2010

D: HCM=hypertrophic cardiomyopathy; DCM=dilated cardiomyopathy; HF=heart failure; AMI=acute myocardial infarction; CAD=coronary artery disease; CVD= cardiovascular diseases; AF=atrial fibrillaton;

creatin kinase MB; cTnI=cardiac troponin I; PCR=polymerase chain reaction; RT-qPCR=quantitative real-time PCR; IHC=immunohistochemistry; WB=western blotting.

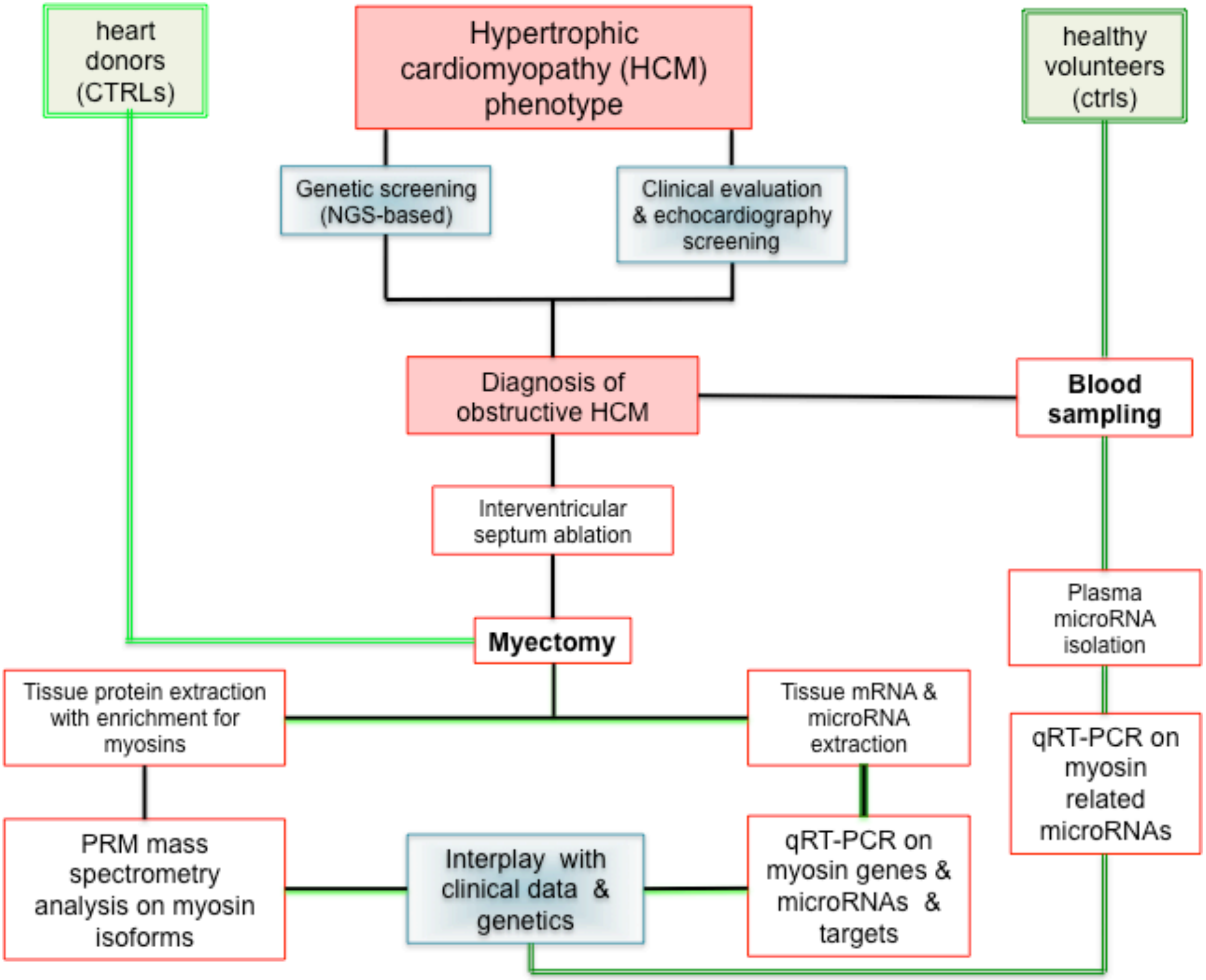

Fig. S1. Flowchart of the experimental study

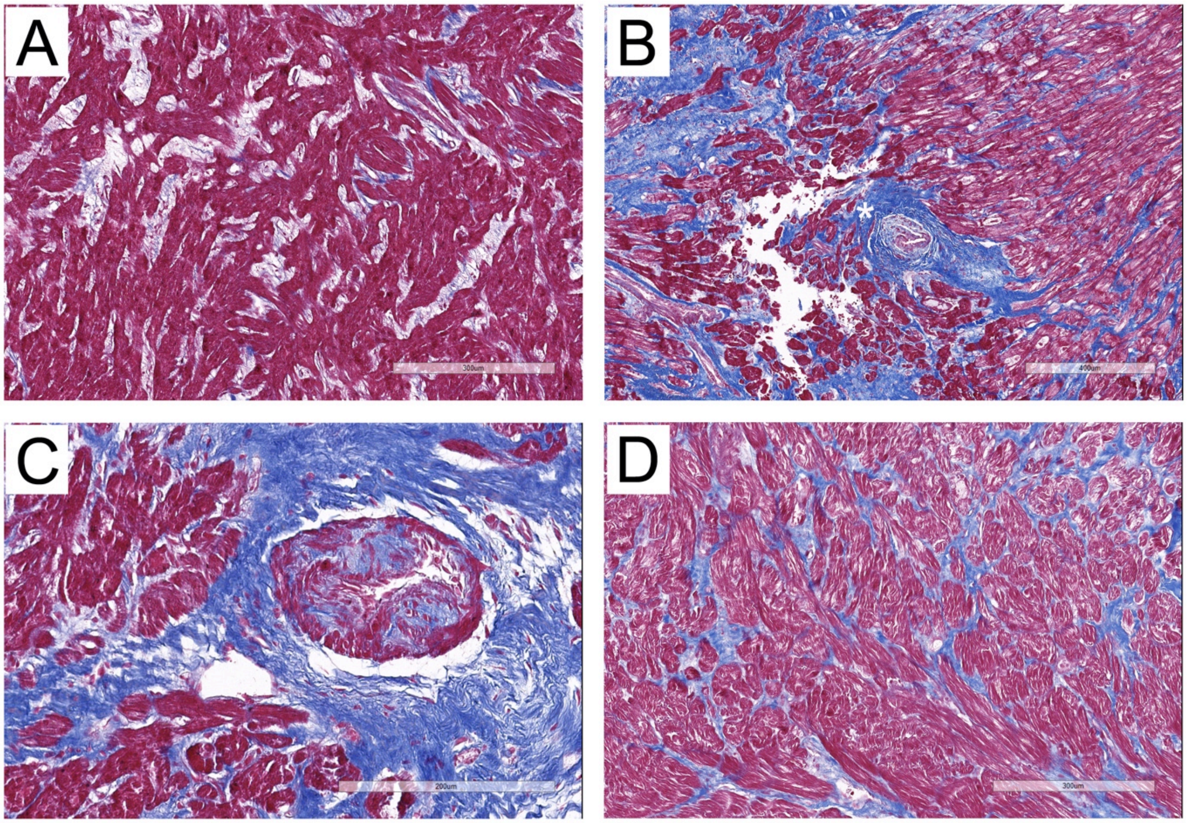

Fig. S2 Histology of HCM myectomy samples. Representative images from paraffin sections demonstrating the main features of HCM are presented. Specifically: cardiomyocytes with hypertrophy and disarray (A), an area of microscar adjacent to a remodeled coronary arteriole (asterisk) (B), a coronary arteriole with medial hypertrophy and fibrosis, and luminal stenosis (C), myofiber disarray in a context of interstitial fibrosis (D) are shown. Scale bars indicate the magnification.

A

	Immunogen		Sequence homology with			Assay reactivity: Cross-reactivity with			
	TYPE	Aminoacids in the protein sequence	MYH6, UniProt ID P13533	MYH7, UniProt ID P12883	MYH7B, UniProt ID A7E2Y1	MYH6, UniProt ID P13533	MYH7, UniProt ID P12883	MYH7B, UniProt ID A7E2Y1	NOTES
Human Myosin Heavy Chain 6, Cardiac Muscle, Alpha (MYH6) ELISA Kit MBS451509, MyBioSource	C-terminal of the human protein	1742-1939	/	98%	77%	/	70-85%	<30%	Tested by the company
Myosin Heavy Chain 7, Cardiac Muscle, Beta (MYH7) ELISA Kit MBS2021497, MyBioSource	human protein	1268-1516	96%	/	70%	none	/	not expected	Tested vs. human MYH6 recombinant protein by the company: no positive signal. Cross-reactivity with MYH7B excluded by the company based on the sequence homology
Myosin Heavy Chain 7B, Cardiac Muscle Beta (MYH7B) ELISA Kit MBS2020415, MyBioSource	human protein	81-300	65%	63%	/	not expected	not expected	/	Cross-reactivity withMYH6 and MYH7 excluded by the company based on the sequence homology

B

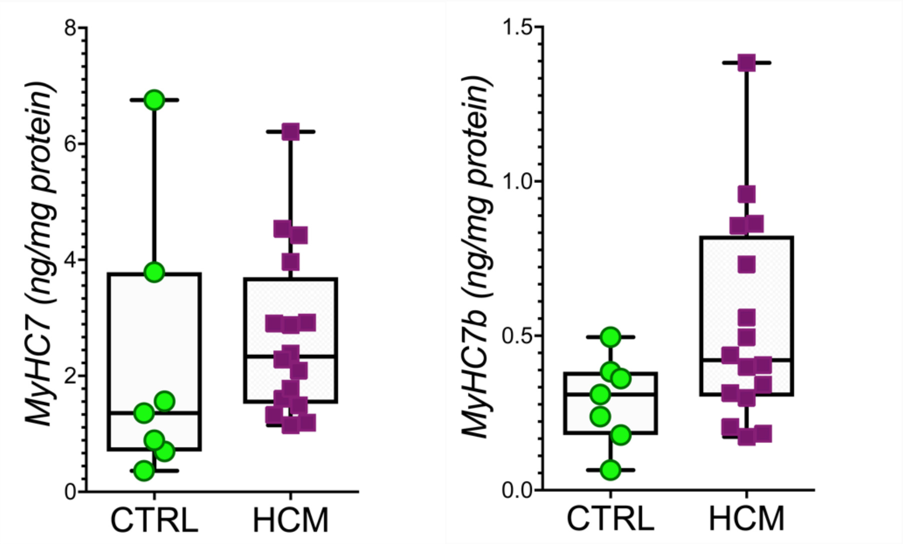

Fig. S3. Analysis of Myosin heavy chain isoforms by ELISA. Analysis on selected ELISA kit immunogens is shown in the Table (A), underlying the sequence homology between protein isoform immunogens and the possible bias due to cross-reactivity. The company kindly provided information on tests specificity and limitations (see notes). Results obtained from myectomy extracts from HCM patients and donor hearts (CTRL) for MyHC7 and MyHC7B by the same ELISA assays listed in **A** are shown as dots into boxes (min to max) and display no significant difference between groups.

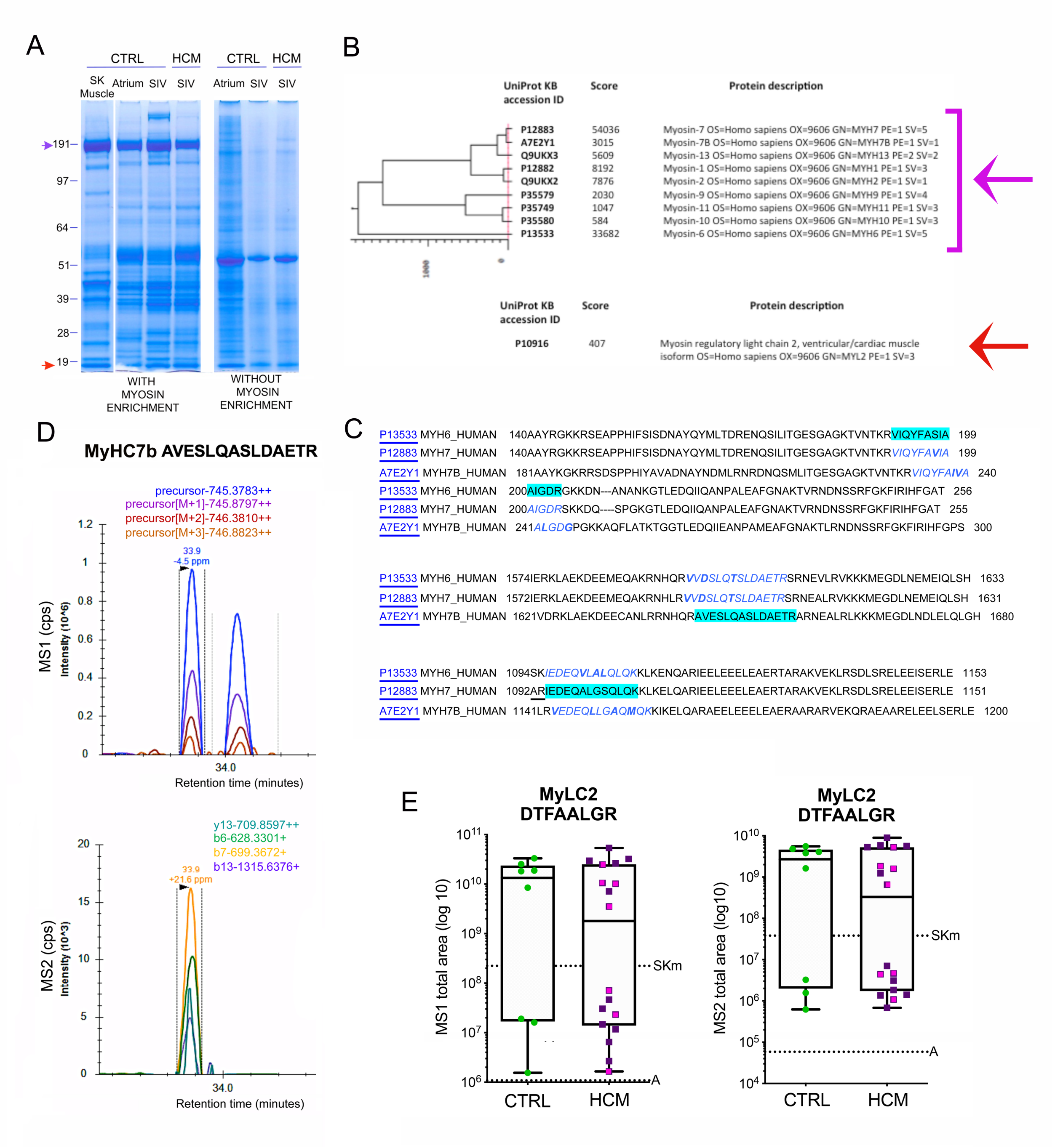

Fig. S4. Analysis of Myosin heavy chain isoforms by mass spectrometry. Representative Coomassie-stained SDS PAGE gel comparing standard protein extraction in RIPA buffer vs. extraction with myosin-enrichment is shown. The same cardiac samples are run in both conditions. Stronger MyHCs band is found close to marker line 191kDa (violet arrow) in enriched samples, that for MyLC2 at 19kDa (red arrow) (**A**). Mascot software search results show the first protein family hit by Mascot search on the band at 191-kDa

molecular weight as hierarchically clustered dendrogram. Each identified myosin isoform is listed. The Mascot search results on the band at 19 kDa corresponding to MyLC2 are also reported. UniProtKB accession IDs and scores are listed (**B**).
Representative ion chromatograms for both MS1 (precursors) and MS2 (fragments) signals from a unique tryptic peptide from MyHC7b**,** selected and analyzed by PRM (Parallel Reaction Monitoring) in MS are shown. Sequence, retention time (minutes), mass error (ppm), m/z, charge and type of fragments are indicated (**C**).
Representative MyHC peptides analyzed by PRM in MS and showed in Figure 1 are evidenced in azure. The aligned partial sequences of the other MyHCs are also presented (blue characters) and the aminoacids differing from those in the analyzed peptide indicated by bold letters (**D**). PRM quantification of MyLC2 in HCM (n=19) and CTRL (n=8) is presented as MS1 and MS2 total area of the selected peptide (**E**). Mean values from 4 technical replicates/sample are presented as dots into boxes (min to max).

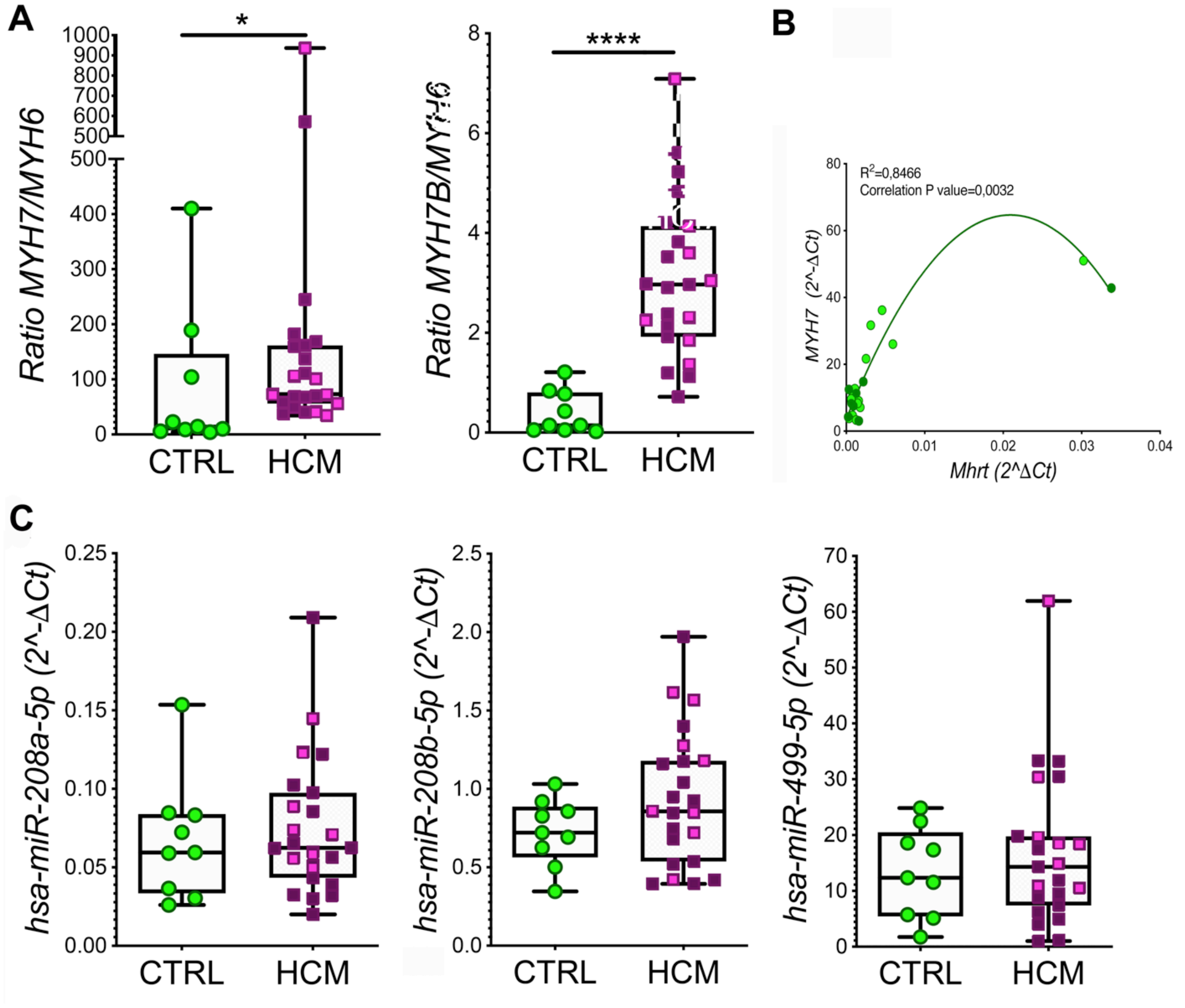

Fig. S5. **Expression of *MYH and MyomiRs in the myocardium*.** The ratios *MYH7/MYH6* and *MYH7B/MYH6* expressed genes evaluated by RT-PCR are shown (**A**), supporting the switch from fast to slow myosins at gene level in HCM vs. CTRL. The correlation between MYH7 expressed gene and the encoded lncRNAs Mhrt is presented (**B**). The expression levels of *miR-208a*, *miR-208b* and *miR-499* evaluated by RT-qPCR in the myocardium of HCM vs. CTRL are shown, displaying a weak relation between myocardial *miR-499* expression and mutation presence (**C**).

Interaction

MYH6 *[ENSP00000386041]*	↔	MYH7 *[ENSP00000347507]*
Myosin-6; Muscle contraction; Belongs to the TRAFAC class myosin-kinesin ATPase superfamily. Myosin family		Myosin-7; Myosins are actin-based motor molecules with ATPase activity essential for muscle contraction. Forms regular bipolar thick filaments that, together with actin thin filaments, constitute the fundamental contractile unit of skeletal and cardiac muscle; Belongs to the TRAFAC class myosin-kinesin ATPase superfamily. Myosin family

Evidence suggesting a functional link:

Neighborhood in the Genome:	none / insignificant.	
Gene Fusions:	none / insignificant.	
Cooccurence Across Genomes:	none / insignificant.	
Co-Expression:	yes (score 0.333). In addition, putative homologs are coexpressed in other species (score 0.062).	Show
Experimental/Biochemical Data:	none, but putative homologs were found interacting in other species (score 0.737).	Show
Association in Curated Databases:	yes (score 0.900).	Show
Co-Mentioned in PubMed Abstracts:	yes (score 0.589). In addition, putative homologs are mentioned together in other species (score 0.163).	Show
Combined Score:	0.981	

Note: the two proteins have some sequence similarity (3662.1 bits over 1929 amino acids. click here to see the alignment).

Predictions for specific actions:

Binding:	yes (score: 0.965)	Show

Interaction

MYH7 *[ENSP00000347507]*	↔	MYH7B *[ENSP00000262873]*
Myosin-7; Myosins are actin-based motor molecules with ATPase activity essential for muscle contraction. Forms regular bipolar thick filaments that, together with actin thin filaments, constitute the fundamental contractile unit of skeletal and cardiac muscle; Belongs to the TRAFAC class myosin-kinesin ATPase superfamily. Myosin family		Myosin-7B; Involved in muscle contraction; Myosin heavy chains

Evidence suggesting a functional link:

Neighborhood in the Genome:	none / insignificant.	
Gene Fusions:	none / insignificant.	
Cooccurence Across Genomes:	none / insignificant.	
Co-Expression:	yes (score 0.069). In addition, putative homologs are coexpressed in other species (score 0.120).	Show
Experimental/Biochemical Data:	none / insignificant.	
Association in Curated Databases:	yes (score 0.540).	Show
Co-Mentioned in PubMed Abstracts:	yes (score 0.537). In addition, putative homologs are mentioned together in other species (score 0.085).	Show
Combined Score:	0.595	

Note: the two proteins have some sequence similarity (2763.4 bits over 1921 amino acids. click here to see the alignment).

Predictions for specific actions:

Binding:	yes (score: 0.577)	Show

Interaction

SOX6 *[ENSP00000379644]*	↔	MYH7 *[ENSP00000347507]*
Transcription factor SOX-6; Transcriptional activator. Binds specifically to the DNA sequence 5'-AACAAT-3'. Plays a key role in several developmental processes, including neurogenesis and skeleton formation; SRY-boxes		Myosin-7; Myosins are actin-based motor molecules with ATPase activity essential for muscle contraction. Forms regular bipolar thick filaments that, together with actin thin filaments, constitute the fundamental contractile unit of skeletal and cardiac muscle; Belongs to the TRAFAC class myosin-kinesin ATPase superfamily. Myosin family

Evidence suggesting a functional link:

Neighborhood in the Genome:	none / insignificant.	
Gene Fusions:	none / insignificant.	
Cooccurence Across Genomes:	none / insignificant.	
Co-Expression:	none, but putative homologs are coexpressed in other species (score 0.062).	Show
Experimental/Biochemical Data:	none / insignificant.	
Association in Curated Databases:	none / insignificant.	
Co-Mentioned in PubMed Abstracts:	yes (score 0.354). In addition, putative homologs are mentioned together in other species (score 0.203).	Show
Combined Score:	0.474	

Interaction

MYH6 *[ENSP00000386041]*	↔	SOX6 *[ENSP00000379644]*
Myosin-6; Muscle contraction; Belongs to the TRAFAC class myosin-kinesin ATPase superfamily. Myosin family		Transcription factor SOX-6; Transcriptional activator. Binds specifically to the DNA sequence 5'-AACAAT-3'. Plays a key role in several developmental processes, including neurogenesis and skeleton formation; SRY-boxes

Evidence suggesting a functional link:

Neighborhood in the Genome:	none / insignificant.	
Gene Fusions:	none / insignificant.	
Cooccurence Across Genomes:	none / insignificant.	
Co-Expression:	none, but putative homologs are coexpressed in other species (score 0.062).	Show
Experimental/Biochemical Data:	none / insignificant.	
Association in Curated Databases:	none / insignificant.	
Co-Mentioned in PubMed Abstracts:	yes (score 0.423). In addition, putative homologs are mentioned together in other species (score 0.094).	Show
Combined Score:	0.466	

Interaction

MYH7 *[ENSP00000347507]*	↔	MYL2 *[ENSP00000228841]*
Myosin-7; Myosins are actin-based motor molecules with ATPase activity essential for muscle contraction. Forms regular bipolar thick filaments that, together with actin thin filaments, constitute the fundamental contractile unit of skeletal and cardiac muscle; Belongs to the TRAFAC class myosin-kinesin ATPase superfamily. Myosin family		Myosin regulatory light chain 2, ventricular/cardiac muscle isoform; Contractile protein that plays a role in heart development and function (By similarity). Following phosphorylation, plays a role in cross-bridge cycling kinetics and cardiac muscle contraction by increasing myosin lever arm stiffness and promoting myosin head diffusion; as a consequence of the increase in maximum contraction force and calcium sensitivity of contraction force. These events altogether slow down myosin kinetics and prolong duty cycle resulting in accumulated myosins being cooperatively recruited to actin [...]

Evidence suggesting a functional link:

Neighborhood in the Genome:	none / insignificant.	
Gene Fusions:	none / insignificant.	
Cooccurence Across Genomes:	none / insignificant.	
Co-Expression:	yes (score 0.426). In addition, putative homologs are coexpressed in other species (score 0.114).	Show
Experimental/Biochemical Data:	yes (score 0.800). In addition, putative homologs were found interacting in other species (score 0.265).	Show
Association in Curated Databases:	yes (score 0.800).	Show
Co-Mentioned in PubMed Abstracts:	yes (score 0.810). In addition, putative homologs are mentioned together in other species (score 0.145).	Show
Combined Score:	0.996	

Predictions for specific actions:

Binding:	yes (score: 0.846)	Show
Catalysis:	yes (score: 0.265)	Show
Reaction:	yes (score: 0.265)	Show

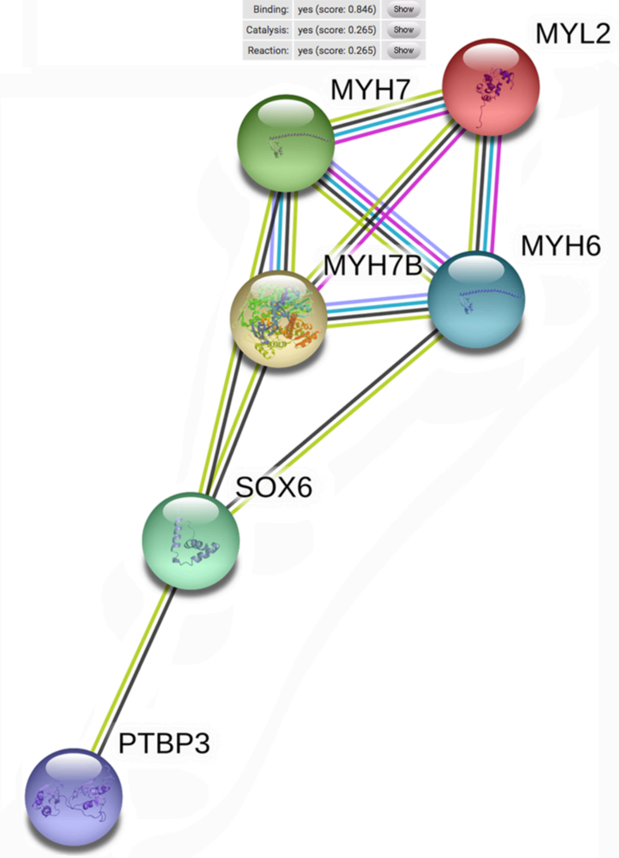

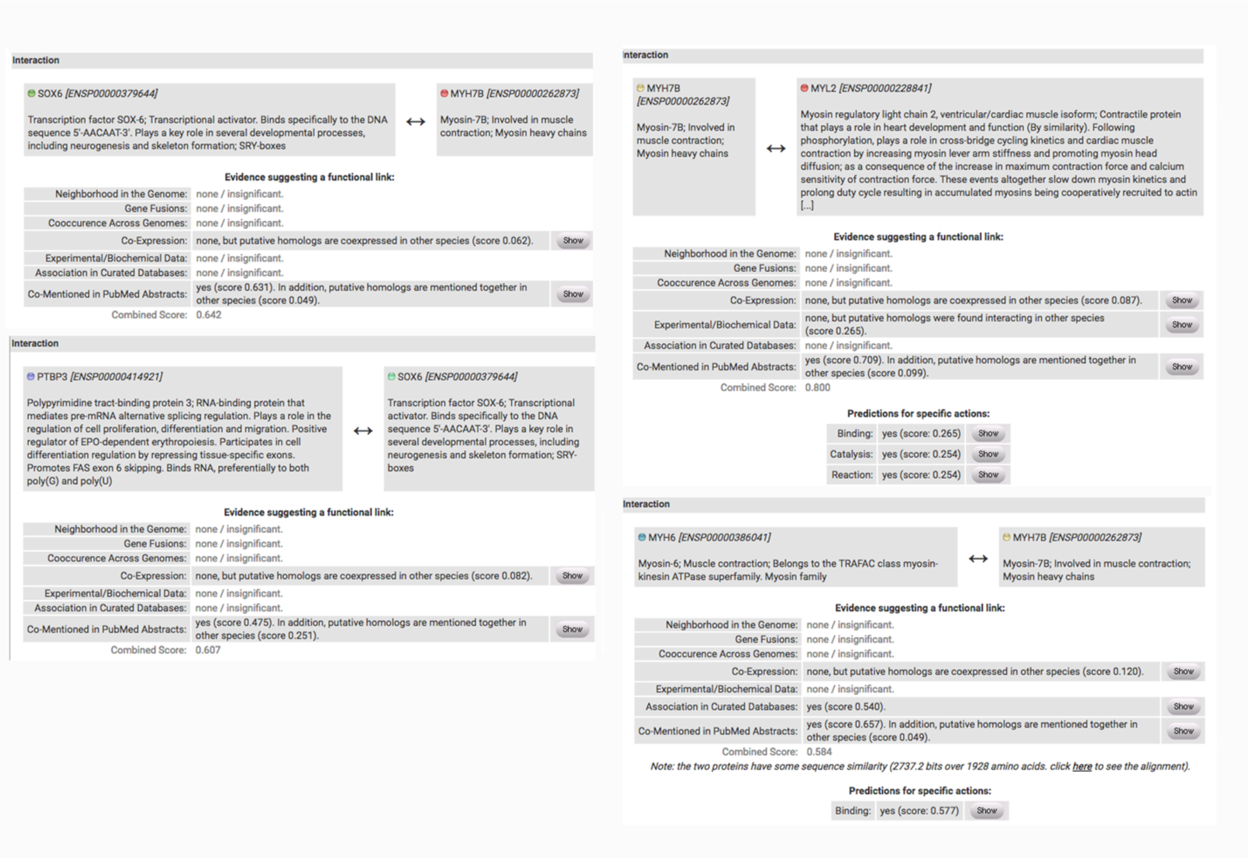

Fig. S6. ***In silico* analysis of protein interactions.** *In silico* analysis by STRING v11 engine of the Interactions among the proteins investigated in the study.

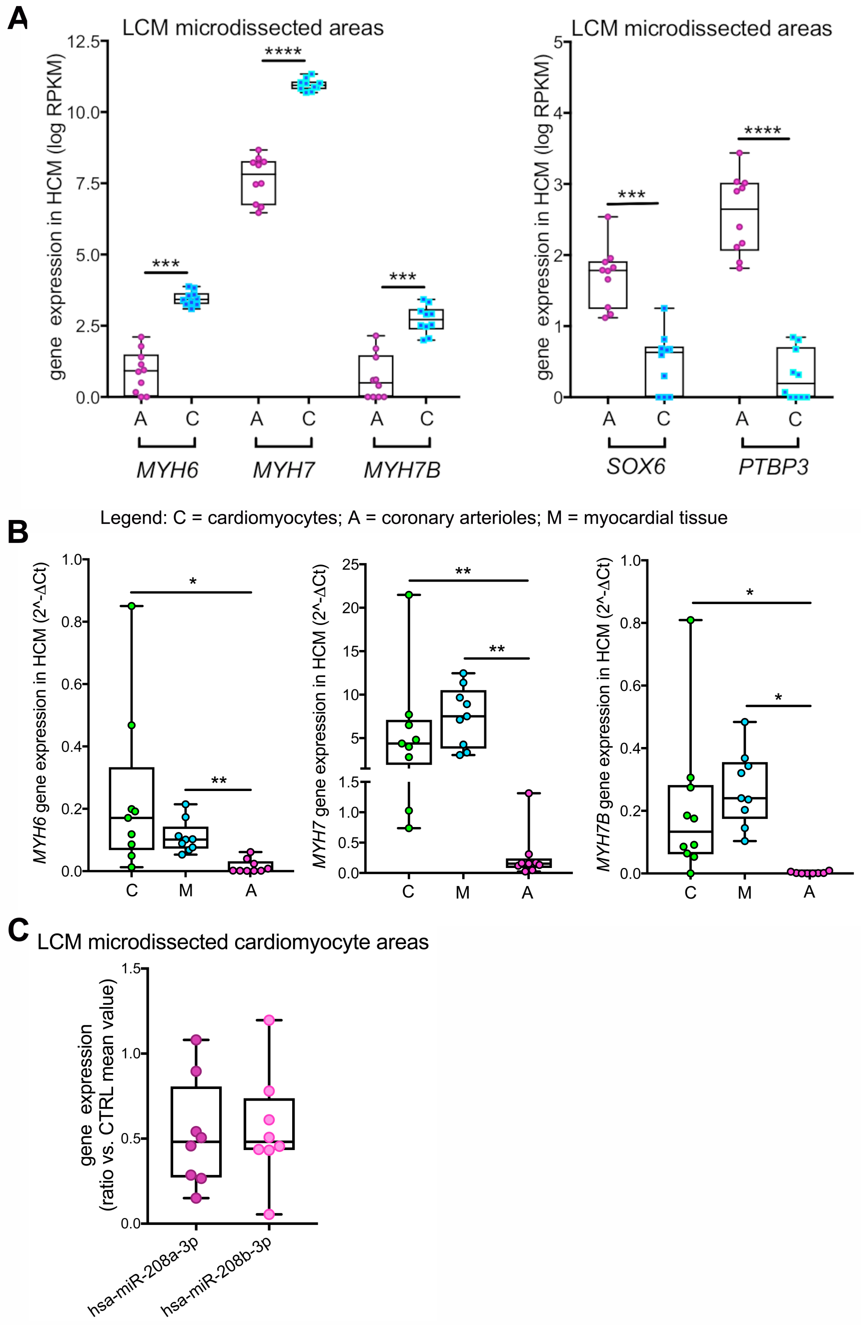

Fig. S7. **Gene Expression in HCM samples submitted to LCM microdissection.**

MYHs, *SOX6* and *PTBP3* expression by cardiomyocytes and coronary arteriole-containing areas of myocardial tissue from HCM patients by NGS is shown (**A**). The MYHs expression in cardiomyocytes, coronary arteriole-containing areas was compared to that of myocardial tissue (**B**). The expression level of MyomiRs *mir-208a* and *miR-208b* in cardiomyocytes from HCM is plotted (**C**).

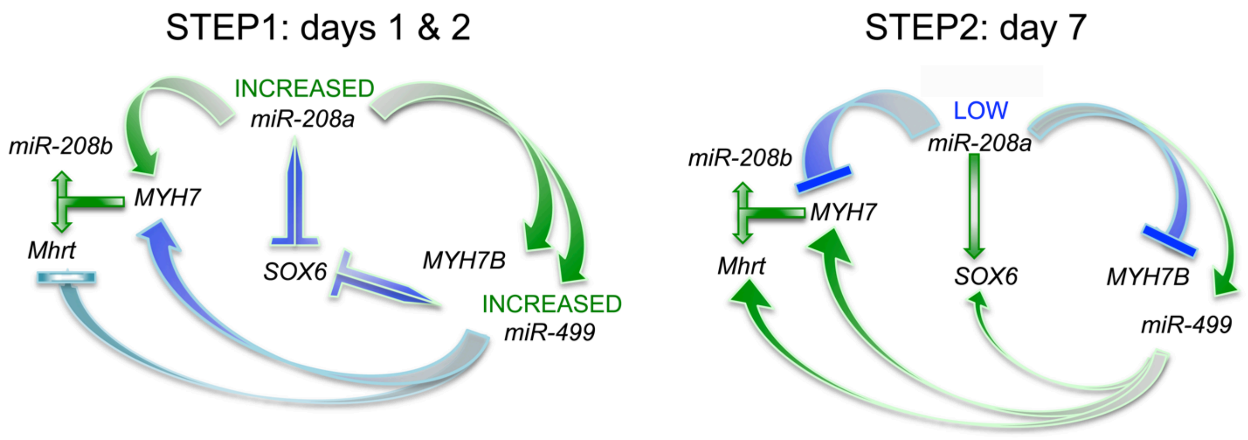

Fig. S8 Schematic representation. The interrelationship between MYHs, MyomiRs, and a target gene found in iPSC- derived cardiomyocytes transfected with miR-208a or miR-499 is summarized, modifying the schematization of MyomiR network proposed by the McCarthy, 2009 (DOI: 10.1152/physiolgenomics.00042.2009)

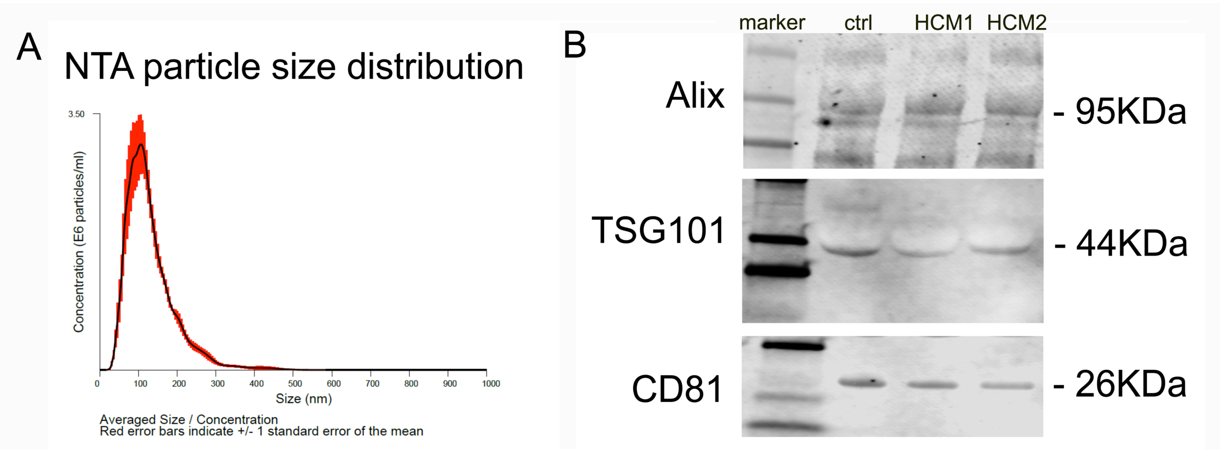

Fig. S9 Exosome characterization Dimensions of vesicles (EV in the manuscript) in the human plasma measured by Nanosight are shown in a representative histogram (**A**). Expression of the exosomal markers ALG-2 interacting protein X (Alix), tumor susceptibility gene 101(TSG101) and tetraspanin CD81 by EV in samples from 1 healthy volunteer and 2 patients with HCM is displayed in western blot images (**B**).
